# Size matters for single-cell C_4_ photosynthesis in *Bienertia*

**DOI:** 10.1093/jxb/erw374

**Published:** 2016-10-12

**Authors:** Ivan Jurić, Vinicio González-Pérez, Julian M Hibberd, Gerald Edwards, Nigel J Burroughs

**Affiliations:** 1 Warwick Systems Biology Centre, University of Warwick, Coventry, UK; 2 Institute of Physics, Bijenička c., Zagreb, Croatia; 3 Department of Plant Sciences, University of Cambridge, Cambridge, UK; 4 School of Biological Sciences, Washington State University, Pullman, WA, USA

**Keywords:** Bienertia, C_4_ photosynthesis, carbon fixation, photon cost, photosynthetic efficiency, single-cell C_4_, spatial modelling

## Abstract

*Bienertia cycloptera* belongs to a diverse set of plants, recently discovered to perform C_4_ photosynthesis within individual mesophyll cells. How these plants accomplish high photosynthetic efficiency without adopting Kranz anatomy remains unanswered. By modelling the processes of diffusion, capture, and release of carbon dioxide and oxygen inside a typical *Bienertia* mesophyll cell geometry, we show that a spatial separation as low as 10 μm between the primary and the secondary carboxylases, can, on its own, provide enough diffusive resistance to sustain a viable C_4_ pathway at 20 °C, with a CO_2_ leakage <35%. This critical separation corresponds to a cell diameter of 50 μm, consistent with the observed range where *Bienertia*’s mesophyll cells start to develop their characteristic mature anatomy. Our results are robust to significant alterations in model assumptions and environmental conditions, their applicability extending even to aquatic plants.

## Teaser

A spatial separation of 10 µm between primary and secondary carboxylases is sufficient for a single-cell C_4_ pathway, even in the absence of any intracellular diffusion barriers.

## Introduction

An excellent example of convergent evolution is the development of the C_4_. photosynthetic pathway in multiple plant genera (Sage, 2004; Sage *et al.*, 2011, 2012). The primary carbon-fixing enzyme in plants, Ribulose-1,5-biphosphate-carboxylase-oxygenase (or Rubisco, for short) can both carboxylate ribulose-1,5-biphosphate (RuBP) with CO_2_ and oxygenate it with O_2_. Carboxylation of RuBP is one of the steps in the Calvin–Benson cycle, also referred to as C_3_ photosynthesis. In contrast, the oxygenation reaction is detrimental, leading to a costly RuBP salvage process (termed photorespiration). Rubisco is not very discriminating with respect to the two atmospheric gases (Farquhar *et al.*, 1980; Zhu *et al.*, 2008), which, combined with the high levels of O_2_ relative to CO_2_ in the atmosphere, hinders the efficiency of photosynthesis. The C_4_ pathway circumvents Rubisco’s poor specificity by placing the enzyme in a CO_2_-rich environment that is actively maintained by means of a chemical CO_2_ pump, which we shall refer to as the C_4_ pump. The pump involves a number of enzymes that capture atmospheric CO_2_ and store it temporarily in four-carbon dicarboxylic acids (malate and aspartate) which diffuse to the compartment where Rubisco is located. Here they are decarboxylated, releasing CO_2_ (Jenkins *et al.*, 1989; von Caemmerer and Furbank, 2003). This is an active process that consumes ATP. The C_4_ pathway, which evolved multiple times in terrestrial plants, is often associated with the development of Kranz anatomy (Sage, 2004). The fixation of atmospheric CO_2_ into C_4_ acids occurs in the mesophyll, while the bundle sheath cells, which harbour Rubisco-rich chloroplasts, are regions where CO_2_ is concentrated. Kranz anatomy thus separates the CO_2_-absorbing and releasing components of the C_4_ pump with multiple diffusion barriers (cell walls and plasma membranes) (von Caemmerer and Furbank, 2003). The presence of such diffusive barriers was considered crucial for an efficient C_4_ pathway, and Kranz anatomy was commonly viewed as a necessary and a defining property of a functional C_4_ plant (von Caemmerer *et al.*, 2014).

**Fig. 1.**
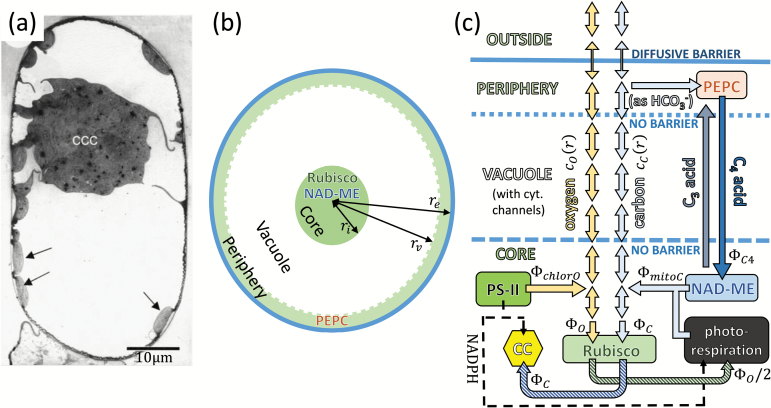
Modelling a *Bienertia* mesophyll cell. (a) Micrograph of a mature *Bienertia* mesophyll cell, taken from Voznesenskaya *et al.* (2002) with permission. The arrows point to the peripheral chloroplasts. (b) Model of a *Bienertia* cell, showing the three compartments, and marking the location of various enzymes. The compartment’s radii, *r*_i_, *r*_v_, and r_e_ are varied in the model, so the picture should not be taken to scale. (c) Abstract schematic of reactions and flows in different spatial regions considered in the model. Yellow arrows represent the oxygen current, and light blue is the CO_2_ current (two-headed arrows represent diffusion). Other arrows represent the C_3_ and C_4_ acid currents (grey and dark blue), and the photorespiratory and Calvin–Benson cycle carbon currents (striped green and blue). The thin dashed line is the NADPH current originating from the Hill reaction in the core’s chloroplasts; it couples the photorespiratory and the Calvin–Benson cycle activity to the oxygen production. The boundary between the periphery and the outside is the only barrier to gas diffusion in the model.

Discovery of terrestrial C_4_ plants (in the Chenopodiaceae family) that lack Kranz anatomy and instead perform all the steps of the C_4_ pathway within the confines of an individual mesophyll cell disproved this view (Voznesenskaya *et al.*, 2001, 2002). In the genus *Bienertia* (*B. cycloptera*, *B. sinuspersici*, and *B. kavirense*), mature chlorenchyma cells are unusually large (80–110 µm in the major dimension; Akhani *et al.*, 2005, 2012; von Caemmerer *et al.*, 2014) and possess a peculiar cellular architecture. The majority of the cell’s chloroplasts are located in a sphere of 20–32 µm in diameter (Akhani *et al.*, 2005, 2012; von Caemmerer *et al.*, 2014), which is positioned in the centre of the cell, Fig. 1a. This ‘central chloroplast compartment’ (CCC) is surrounded by a large vacuole. A portion of the cell’s chloroplasts (referred to as ‘peripheral’) are scattered along the cell’s surface, separated from the CCC by the vacuole. Narrow cytoplasmic channels cross the vacuole connecting the central compartment with the periphery (Voznesenskaya *et al.*, 2005). The C_4_ pump in *Bienertia* functions by shuttling aspartate (a C_4_ acid) and alanine (a C_3_ acid) between the two domains (Voznesenskaya *et al.*, 2005; Offermann *et al.*, 2011), characteristic of NAD-malic enzyme (NAD-ME) type C_4_ plants. Conversion between alanine and aspartate goes through several C_3_ and C_4_ intermediaries (for full details, see, for example, von Caemmerer and Furbank, 2003). The initial carbon capture occurs in the peripheral cytoplasm where carbonic anhydrase (CA) converts CO_2_ to bicarbonate, which is used by phospho*enol*pyruvate carboxylase (PEPC) to turn phospho*enol*pyruvate (PEP, a C_3_ acid) into oxaloacetate (a C_4_ acid). Subsequent release of CO_2_ occurs in the mitochondria within the CCC, where malate (a C_4_ acid) is decarboxylated by NAD-ME. The chloroplasts in the central compartment are filled with Rubisco, which assimilates released CO_2_ (Voznesenskaya *et al.*, 2005; Offermann *et al.*, 2011). A C_4_ pump is thus established between the cell’s periphery and its centre.

The energy from photons absorbed by the chloroplast photosystems is used to produce the ATP and NADPH needed to support carbon assimilation. In C_3_ plants, this includes requirements to support the Calvin–Benson cycle, and photorespiration, while C_4_ plants also need to support the C_4_ cycle. The efficiency of the C_4_ pathway depends on the balance between this additional energy cost and the reduced photorespiration cost. In C_3_ plants, the assimilation cost rises with increasing resistance to diffusion of CO_2_ from the atmosphere to Rubisco (e.g. by stomatal limitation or reduced conductance of CO_2_ from the intercellular air space to Rubisco in photosynthetic cells), or with increasing temperature [when Rubisco’s specificity decreases (Boyd *et al.*, 2015)]. In C_4_ photosynthesis the gradient of CO_2_ concentration is reversed, and the resistance to gas diffusion now benefits the plant. Still, a degree of CO_2_ leakage is inevitable, and a C_4_ pump must run faster than CO_2_ fixation by Rubisco. The fraction of pumped CO_2_ leaking out of the Rubisco-containing compartment ranges from 20% to 40% in plants with Kranz anatomy (Kubásek *et al.*, 2007; von Caemmerer *et al.*, 2014). In plants with a single-cell C_4_ pathway, there are few physical barriers between the locations of the initial carbon capture and CO_2_ release (only a couple of intracellular lipid bilayer membranes), so one would expect a significantly—even prohibitively—higher CO_2_ leakage (von Caemmerer, 2003). Yet studies indicate that the leakage is comparable with that of plants with Kranz anatomy (King *et al.*, 2012; von Caemmerer *et al.*, 2014; Stutz *et al.*, 2014). This raises the question of how single-cell C_4_ plants achieve this apparently high efficacy.

Herein we investigate the efficacy of the C_4_ pathway in *Bienertia* through construction of a spatial mathematical model of a *Bienertia* mesophyll cell. We concentrate on the role of its specific cellular architecture, and, in particular, on the effect that the spatial separation between the periphery and the central compartment has on the efficiency of the C_4_ pathway. Our model goes beyond the previous compartmental models of carbon fixation in plants that tend to oversimplify the spatial aspects of photosynthetic processes (von Caemmerer, 2003, 2013; von Caemmerer *et al.*, 2014). We show that spatial separation alone can act as an effective diffusion barrier provided the cell is larger than a certain critical size.

## Model description

Full details of the model, including more detailed justifications of the underlying assumptions can be found in the Supplementary Model at *JXB* online. In the following, we provide a brief decription of its main features. Our model focuses exclusively on cell processes that involve CO_2_ and O_2_, namely their absorption, production, and diffusion. We look at a single spherical *Bienertia* mesophyll cell, formed of three concentric compartments (Fig. 1b): ‘the core’, ‘the vacuole’, and ‘the periphery’. Their sizes are defined by their radii, *r*_i_, *r*_v_, and *r*_e_, respectively. We model these regions as follows:

(i) The core region (i.e. the CCC) is composed of mitochondria and Rubisco-rich chloroplasts. CO_2_ is produced in the mitochondria through decarboxylation of malate by NAD-ME and as a photorespiratory by-product. We assume little or no CA in the core cytoplasm (Offermann *et al.*, 2015), so CO_2_–bicarbonate conversion can be neglected. CO_2_ can diffuse out of the core region, or react with Rubisco (active site concentration *c*_R_), which we assume is always primed with RuBP and activated. O_2_ is also produced in the core by PSII in the core chloroplasts, and can react with Rubisco, triggering the photorespiratory cycle reactions.
(ii) The vacuole combines both the tonoplast interior and the cytoplasmic channels. CO_2_ and O_2_ diffuse freely here, and no reactions take place.
(iii) The periphery cytoplasm is rich in CA and PEPC (active site concentration *c*_P_) (Voznesenskaya *et al.*, 2002; Offermann *et al.*, 2015). Inorganic carbon will predominantly be in bicarbonate form, which is used by PEPC to carboxylate PEP. Diffusion of CO_2_ and O_2_ between the surrounding airspace and the cell periphery is hampered by the cell wall and membrane (permeability σ_B_), which form the only diffusion barrier in the model.

Although the cell is conceptually divided into three distinct spatial compartments, no intracellular diffusion barriers are placed between these regions. This is a deliberate choice to test the viability of the C_4_ pump when there is nothing but a spatial separation to provide diffusive resistance to gases in the liquid phase. We note that *Bienertia* mesophyll cells are not actually spherical. However, since we are interested in the general effects of size on the efficiency of a C_4_ pathway, a simpler model (which is also more amenable to numerical investigation) will suffice.

The enzymatic reactions follow Michaelis–Menten kinetics. Since detailed kinetic data for *Bienertia*’s Rubisco are not presently available, we use kinetic parameters for maize (*Zea mays*) (Cousins *et al.*, 2010), a well-studied C_4_ plant. When assessing temperature dependence, we also use the data for another C_4_ plant, *Setaria viridis* (Boyd *et al.*, 2015), to infer Rubisco’s temperature response. PEPC and NAD-ME kinetic parameters are taken from *Z. mays* and *Arabidopsis thaliana*, respectively (Kai *et al.*, 1999; Tronconi *et al.*, 2008). Values of all the parameters are listed in Table 1 and their temperature dependence in Supplementary Table S1.

**Table 1.** Parameter values used for modelling C_4_ photosynthesis at 20 °C

Description	Symbol	Units	Value
Rubisco active sites concentration in the core	*c*_R_	mM	Variable
Rubisco carboxylation catalysis rate (Cousins *et al.*, 2010)	*k*_catC_	s^–1^	4.7
Rubisco oxygenation catalysis rate (Cousins *et al.*, 2010)	*k*_catO_	s^–1^	0.49
Rubisco Michaelis concentration for CO_2_ (Cousins *et al.*, 2010)	*K*_C_	µM	16.2
Rubisco Michaelis concentration for O_2_ (Cousins *et al.*, 2010)	*K*_O_	µM	183
PEPC concentration in the periphery	*c*_P_	mM	Variable
PEPC carboxylation catalysis rate (Kai *et al.*, 1999)	*k*_catP_	s^–1^	150
PEPC Michaelis constant for HCO_3_^–^ (Kai *et al.*, 1999)	*K*_P_	µM	100
NAD-ME decarboxylation catalysis rate (Tronconi *et al.*, 2008)	*k*_catN_	s^–1^	37.6
NAD-ME Michaelis constant for malate (Tronconi *et al.*, 2008)	*K*_M_	µM	300
Diffusion constant for carbon dioxide (Mazarei and Sandall, 1980)	*D*_C_	µm^2^ s^–1^	1800
Diffusion constant for oxygen (Mazarei and Sandall, 1980)	*D*_O_	µm^2^ s^–1^	1800
Combined permeability of the cell wall and plasma membrane	σ_B_	µm s^–1^	Variable
Concentration of dissolved CO_2_ in equilibrium with air at 20 °C	*c*_Ceq_	µM	15.4
and standard atmospheric pressure (with 400 ppm of CO_2_) (Carroll *et al.*, 1991)			
Concentration of dissolved O_2_ in equilibrium with air	*c*_Oeq_	µM	284
at 20 °C and standard atmospheric pressure (Murray and Riley, 1969)			
Radius of the core	*r*_i_	µm	Variable
Radius of the vacuole	*r*_v_	µm	Variable
Radius of the cell	*r*_e_	µm	Variable
Base photon cost of RuBP regeneration (Zhu *et al.*, 2010)	φ_C_	1	8
Base photorespiration photon cost (Zhu *et al.*, 2010)	φ_O_	1	9
Base cost of pyruvate-to-PEP conversion (Zhu *et al.*, 2010)	φ_C4_	1	4

The model implicitly assumes that other reactions of the C_4_ cycle, and those involving Rubisco (activation and RuBP binding), as well as the light intake by PSI and PSII, are not rate limiting. This also implies that the base C_3_ substrate (alanine), as well as the C_4_ product (aspartate), are abundant within the cell—a necessary condition for optimal functioning of the C_4_ pathway in any case. We do not model bicarbonate kinetics explicitly, only modelling dissolved CO_2_. This is justified, because HCO_3_^–^ can only equilibrate with CO_2_ in the periphery, where CA is present, and their interconversion elsewhere will be negligible (Heinhorst *et al*., 2006; Johnson, 1982; see Supplementary Model for details).

We solve a set of partial differential equations for the radial concentrations of CO_2_ and O_2_, *c*_C_(*r*) and *c*_O_(*r*), respectively, under steady-state conditions. The C_3_/C_4_ acid currents and the O_2_ production are determined by flux balance conditions. Namely, the CO_2_ production in the core must match the photorespiratory activity and the rate of the PEPC carboxylation in the periphery, while the O_2_ production has to match NADPH requirements of the Calvin–Benson cycle and photorespiration.

The efficiency of carbon fixation is expressed in terms of the photon cost (the inverse of the quantum yield) associated with assimilation. This is the minimal number of photons (measured per carbon atom assimilated) that need to be collected by PSI and PSII to cover the ATP and NADPH requirements of the Calvin–Benson cycle, the photorespiration, and the C_4_ pump operation (Farquhar *et al*., 1980; Zhu *et al*., 2010; Kramer and Evans, 2011). To analyse the effects of cell geometry on the photosynthetic efficacy, we always optimize the C_4_ pump reaction kinetics; that is, for given compartment radii, we find the concentration of PEPC in the periphery for which the photon cost is minimized. If the optimal cost is achieved at a non-vanishing PEPC concentration, we can say that the C_4_ photosynthetic pathway is a viable and preferable alternative to C_3_-only photosynthesis for the selected cell geometry.

We emphasize that the comparison of the C_3_ and C_4_ pathway efficiencies is always made for the *Bienertia*-like cell geometry. The architecture of mesophyll cells in C_3_ plants is different (Tholen and Zhu, 2011), and such mesophyll cells would probably outperform *Bienertia*-like C_3_ cells of equal size. Our aim is instead to find the minimal mesophyll cell size above which a plant using a single-cell C_4_ pump (in *Bienertia*-like cell geometry) is certain to benefit from its use.

## Results

The model has several parameters that can affect the photosynthetic efficiency: the radii of the three compartments, *r*_i_, *r*_v_, and *r*_e_, the PEPC concentration in the periphery, *c*_P_, the concentration of Rubisco active sites in the core, *c*_R_, and the cell barrier (wall and membrane) permeability, σ_B_. To study the model in depth, we initially examine the system’s behaviour at a particular Rubisco concentration and cell barrier permeability. The value of *c*_R_=2 mM is in line with general estimates of the Rubisco active site concentration within chloroplast stroma (2–5 mM) (von Caemmerer, 2000), allowing for the fact that the core also contains mitochondria. Estimates of the permeability of the cell wall and cell membrane vary over two orders of magnitude (Terashima *et al.*, 2006; Evans *et al.*, 2009). We set σ_B_=100 µm s^–1^, but also investigate the effects of varying σ_B_ (and *c*_R_) in a later section. With the PEPC concentration optimized for minimal photon cost, only the geometrical parameters remain. We fix the thickness of the peripheral layer, *r*_e_–*r*_v_ to 5 µm (it should not be much wider than the width of a peripheral chloroplast). Reasonable variation in the peripheral thickness will not affect the results as variation in the PEPC concentration will compensate. Two parameters remain: the core radius, *r*_i_, and the periphery to core distance (i.e. the depth of the surrounding vacuole), *r*_v_–*r*_i_. We investigate *Beinertia*’s assimilation efficiency in a geometry space defined by these two parameters.

### Optimal PEPC concentration and photon cost

**Fig. 2.**
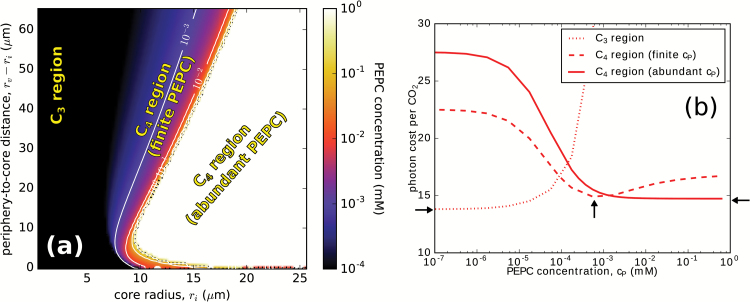
C_4_-optimized PEPC levels. (a) Optimal PEPC concentration as a function of the core radius, *r*_i_, and the periphery to core distance, *r*_v_–*r*_i_, for a cell at 20 °C with Rubisco concentration of *c*_R_=2 mM and cellular barrier permeability of σ_B_=100 μm. Other parameters are as in Table 1. Level lines are in white. The abundant PEPC region, which appears white, has PEPC concentration >1 mM; the black region on the left is the C_3_ region, with zero PEPC concentration. (b) Dependence of the photon cost on PEPC concentration, *c*_P_, at three exemplary points in different regions of (a), with co-ordinates *r*_i_=5.5 μm, 10.5 μm, and 12.6 μm, and *r*_v_–*r*_i_=26 μm. Arrows mark the positions of the photon cost minima (i.e. the optimal PEPC concentrations) in the three cases (discussed in the text).

Figure 2a shows the optimal PEPC concentration, *c*_Popt_ (the concentration at which the photon cost is minimized), as a function of *r*_i_ and *r*_v_–*r*_i_. For corresponding NAD-ME concentrations, see Supplementary Fig. S1. Three distinct regions are visible. Cells with small cores, *r*_i_ ~6 µm, fall in a ‘C_3_ region’, where *c*_Popt_=0, so the system prefers to deactivate the C_4_ pump and function as a C_3_ plant. At the other extreme is the region where the optimal PEPC concentration is unbounded (i.e. the C_4_ cycle works best when PEPC is present in abundance). [Our numerical method can produce a finite PEPC solution for *c*_P_ up to ~2 mM. Where no photon cost minimum is found in this range, we use the abundant-*c*_P_ solution (see Supplementary Model) to evaluate the minimal photon cost.] The photon cost in this case essentially levels off for *c*_P_ >1 mM, so above this value the actual concentration of PEPC will be constrained by other factors. In this ‘abundant-PEPC regime’, the concentration of dissolved inorganic carbon in the periphery will be negligible, as the periphery effectively absorbs all CO_2_ diffusing into it from the cell exterior and the vacuole. Between these two regions is an intermediate regime where the minimal photon cost is found at finite PEPC concentration, *c*_Popt_. Figure 2b shows how the photon cost changes with PEPC concentration in these three regions, with the photon cost minimum at zero, finite, and infinite PEPC concentration, respectively.

**Fig. 3.**
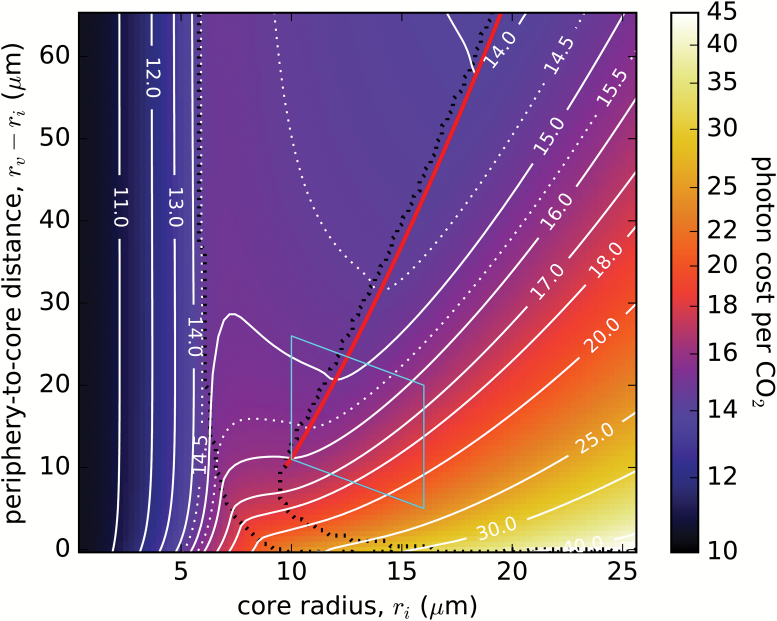
C_4_-optimized photon cost as a function of the core radius, *r*_i_, and the periphery to core distance, *r*_v_–*r*_i_. The lines of constant photon cost are in white. The red line traces the local minima (in *r*_i_) of the C_4_-optimized photon cost. Dashed black lines mark boundaries between regions where the optimum cost is found at zero, finite, and abundant PEPC concentration (compare Fig. 2a, also see the main text). The light-blue parallelogram shows the measured range of a mature *Bienertia* cell and CCC sizes (from Akhani *et al.*, 2005, 2012). Parameters are as in Fig. 2.

**Fig. 4.**
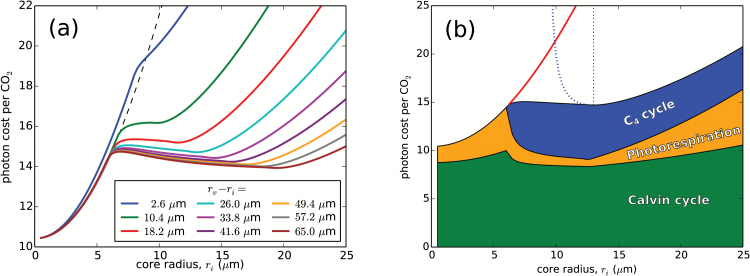
Photon cost variation with core radius. (a) C_4_-optimized photon cost as a function of the core radius, *r*_i_, for a choice of fixed periphery to core distances, *r*_v_–*r*_i_. The dashed black line shows the C_3_-only pathway cost at a medium *r*_v_–*r*_i_ of 18 μm; it has negligible dependence on the periphery to core distance. (b) Breakdown of the C_4_-optimized photon cost into the costs connected with the operation of the Calvin–Benson, the photorespiratory, and the C_4_ cycle (green, orange, and blue), for the line in (a) with *r*_v_–*r*_i_=26 μm. The red line shows the C_3_ pathway photon cost at the same cell geometry. The dotted blue line shows the photon cost when the C_4_ pump runs with PEPC in abundance (in the finite-PEPC region this cost is not optimal). The vertical dotted line marks the position of the optimal-geometry line (the red line in Fig. 3). Parameters are as in Fig. 2.

Figure 3 shows how the C_4_-optimized photon cost (i.e. the minimal cost obtained under optimal C_4_ pump operational conditions) depends on the core radius, *r*_i_, and the periphery to core distance, *r*_v_–*r*_i_. This photon cost ‘landscape’ showcases the main results of this study. In the C_3_ region, the optimal photon cost rises as *r*_i_ increases, due to the increasing ratio of oxygenation to carboxylation as Rubisco becomes starved of CO_2_ in the enlarging core. Around *r*_i_ ~6 μm (the exact point depending on the periphery to core distance), we cross into the C_4_ region and the photon cost starts to decrease as the C_4_ pump becomes operational and its activity (i.e. the PEPC concentration *c*_Popt_) rises. Here we are entering a ‘valley’ in the photon cost landscape—as once we cross into the abundant PEPC region, the photon cost again starts to increase with *r*_i_ due to rising photorespiration (see later). The bottom of this elongated valley (marked by a red line in Fig. 3) roughly coincides with the border between the regions of finite and abundant *c*_Popt_. We shall refer to this valley bottom as the ‘optimal-geometry line’ as it determines the minimal photon cost (and the corresponding optimal core radius) achievable at a particular periphery to core distance (or cell size). The depth and shape of the photon cost valley can be more clearly seen in the constant *r*_v_–*r*_i_ cross-section profiles of the photon cost landscape in Fig. 4. The valley is fairly shallow, but the difference between the C_3_ pathway and the C_4_ pathway costs at the same cell geometry [i.e. at a given point (*r*_i_, *r*_v_–*r*_i_) within the valley] is large. The C_4_-optimized photon cost within the valley is ~3–4 photons per CO_2_ higher when compared with the cost for very small *r*_i_ in the C_3_ region (however, there is a limit to how small a CCC can become, and values of *r*_i_ < 3 μm are not realistic). The valley is not present at small cell sizes (i.e. for small *r*_v_–*r*_i_), but only forms at cell radii *r*_e_ above ~25 μm. We can compare the position of the valley on Fig. 3 with the dimensions of mature *Bienertia* cells and their central compartments. Measured CCC radii range from 10 µm (*B. kavirense*) (Akhani *et al.*, 2012) to 16 µm (*B. sinuspersici*) (Akhani *et al.*, 2005). *Bienertia* cells are only approximately spherical, and their major and minor dimensions can differ substantially. As a representative measure, we take half the largest reported width (26 µm, in *B. cycloptera*; Akhani, *et al.*, 2005) and half the shortest length (41 µm, in *B. kavirense*; Akhani *et al.*, 2012) as our reference cell radii *r*_e_ range. These ranges (light blue parallelogram in Fig. 3) nicely encompass the early part of the optimal-geometry line.

**Fig. 5.**
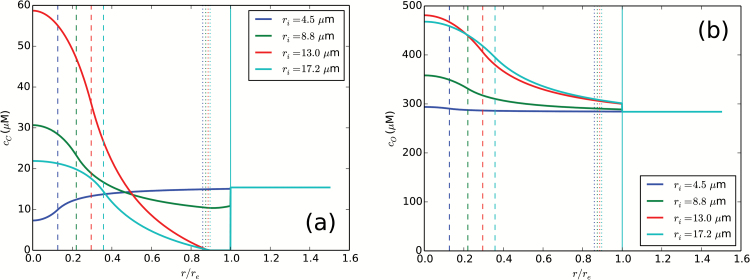
CO_2_ and O_2_ concentration profiles. Radial profiles of carbon dioxide (a) and oxygen (b) concentration for a fixed periphery to core distance, *r*_v_–*r*_i_=26 μm, and varying core radii, *r*_i_. Selected radii correspond to the C_3_ region (*r*_i_=4.5 μm, blue), finite PEPC region (*r*_i_=8.5 μm, green), and abundant PEPC (*r*_i_=12.5 μm, 16.5 μm, red and light-blue respectively). Distances are scaled by the cell radius, *r*_e_. The dashed, dotted, and full vertical lines mark the *r*_i_, *r*_v_, and *r*_e_ radii. Parameters are as Fig. 2.

Typical radial profiles of the CO_2_ and O_2_ concentration in the cell are shown in Fig. 5. A substantial spatial variation in the CO_2_ concentration is visible. In the C_3_ region of the parameter space, CO_2_ is partially depleted in the core. Turning on the C_4_ pump leads to an increase in the core CO_2_ concentration, which can surpass the external dissolved CO_2_ concentration (i.e. the concentration of dissolved CO_2_ at equilibrium with the partial CO_2_ pressure in the surrounding air) by several-fold. On the other hand, the concentration of CO_2_ in the periphery decreases, and the periphery becomes depleted of inorganic carbon when we enter the abundant PEPC regime. The O_2_ concentration also varies spatially, but to a lesser extent. It is highest in the core, where it is produced by the Hill reaction.

### CO_2_ concentration and leakage

**Fig. 6.**
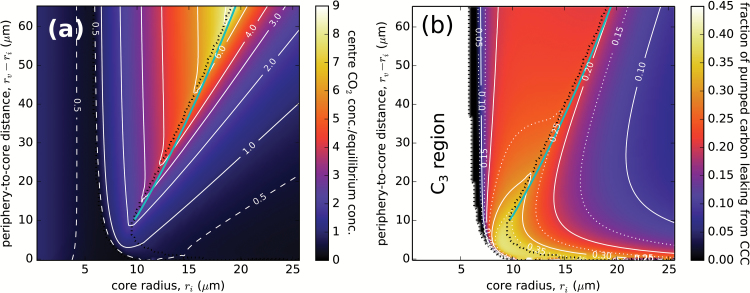
CO_2_ concentration and leakage. (a) Concentration of CO_2_ in the cell centre relative to the external dissolved concentration (i.e. the concentration of dissolved CO_2_ in equilibrium with air at standard atmospheric pressure and 20 °C, *c*_C_ (*r*=0)/*c*_Ceq_, as a function of *r*_i_ and *r*_v_–*r*_i_. (b) CO_2_ leakage from the cell core as a proportion of the C_4_ pump current. The optimal-geometry line is marked light-blue. Other lines are as in Fig. 3. Parameters are as in Fig. 2.

Figure 6a shows the C_4_-optimized CO_2_ concentration at the cell centre, relative to the external dissolved concentration [*c*_C_ (*r*=0)/*c*_Ceq_]), as a function of core radius, *r*_i_, and the periphery to core distance, *r*_v_–*r*_i_. The central CO_2_ concentration is maximal (from 2- to 7-fold higher than the external dissolved CO_2_ concentration) along the finite–abundant PEPC boundary, close to the optimal-geometry line. This is expected, since a high concentration of CO_2_ around Rubisco reduces the photorespiratory losses as O_2_ is outcompeted. However, the high CO_2_ concentration in the centre will also result in increased CO_2_ leakage from the core region, and correspondingly in an increase in the fraction of wasted C_4_ pump cycles (i.e. the futile cycles). The CO_2_ leakage, shown in Fig. 6b, is thus also maximal along the finite–abundant PEPC border, and is highest at small core radii and periphery to core separations.

What determines the finite–abundant PEPC border? Or, why is the C_4_ pump operation ‘scaled back’ in the finite PEPC region? If we decrease the core radius while within the abundant PEPC region, the central CO_2_ concentration will rise, as the release of the carbon collected at the periphery is concentrated in a smaller volume. This quenches photorespiration but incurs increasingly high pump running costs as CO_2_ leakage from the core intensifies. At some critical *r*_*i*_—which defines the finite–abundant PEPC border—the cost of shuttling back leaked carbon cannot be compensated by further reduction in photorespiration, and the C_4_-optimized cost (at smaller *r*_i_) is achieved by throttling down the C_4_ pump (i.e. by lowering the PEPC concentration)—leading to a decrease in the central CO_2_ concentration and reduced leakage. As the activity of the Hill process is dictated by requirements for reducing power, the O_2_ concentration in the centre displays a similar, though less pronounced trend (Supplementary Fig. S2).

The trade-off between lower photorespiration and increased CO_2_ leakage can be best seen in Fig. 4b, which shows the breakdown of the total photon cost into its Calvin–Benson cycle (RuBP regeneration), photorespiration, and C_4_ pump components, along a fixed periphery to core distance (see also Supplementary Fig. S3). Optimization of the photon cost is accomplished by reducing photorespiration—minimized at the finite–abundant PEPC region border—even if it means using up to 40% of the collected photons to run the C_4_ pump. The CO_2_ leakage along the optimal-geometry line does not exceed 36% (Fig. 6b), in accordance with experimental observations (von Caemmerer *et al.*, 2014). Hence, we may conclude that even a modest spatial separation (≥10 µm) between the locations of carboxylation and decarboxylation in an optimized C_4_ pathway suffices to constrain the leakage to acceptable levels (i.e. physical diffusion barriers are not necessary).

### Variations of the model

The findings presented in previous sections are surprisingly robust to changes in the model parameters, and qualitative features generally remain fully preserved. Changing the position of mitochondria within the central compartment (Supplementary Fig. S10), changing the type or concentration of Rubisco in the core region (Supplementary Figs S11, S12), changing the CO_2_ concentration in the surrounding airspace (Supplementary Fig. S13), and even changing the surrounding environment from air to water (Supplementary Fig. S14) merely result in some, mostly minor, changes to the photon cost. These variations are addressed in the Discussion. In the following, we examine the influence of the two parameters that show the most pronounced impact on the photosynthetic efficiency. These are the ambient temperature and the permeability of the cell’s boundary.

#### Varying the ambient temperature

Temperature dependence is relevant because C_4_ photosynthesis is generally—and for *Bienertia* in particular—an adaptation to arid and hot climates. An increase in temperature causes a rise in Rubisco activity, but reduces its carboxylation to oxygenation specificity, making carbon-concentrating mechanisms all the more beneficial (Boyd *et al.*, 2015). Many parameters used in the model will change with temperature. Unfortunately, in many cases, the temperature dependence is unknown. We use reasonable (but tentative) conjectured temperature dependencies. These are provided, together with their justification, in Supplementary Table S1.

**Fig. 7.**
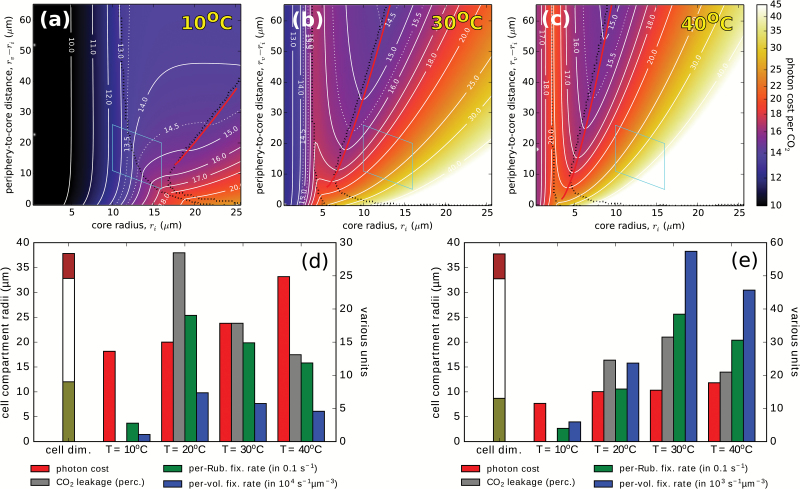
Temperature dependence of C_4_ photosynthesis. (a–c) C_4_-optimized photon cost landscapes at ambient temperatures of 10, 30, and 40 °C, plotted as Fig. 3 (20 °C). (d) and (e) Comparisons of the CO_2_ leakage, photon cost, and assimilation rates at two fixed cell geometries at various temperatures. The three compartment radii in (d) and (e) are marked on the left-most tricolour meters, and can be read on the left *y*-axis. The values of other quantities are to be read on the right *y*-axis. The comparison in (d) is taken at a point in the photon cost landscape that lies on the optimal-geometry line at 20 °C, with a photon cost (at 20 °C) of 15 photons per CO_2_. The comparison in (e) is taken at a point that lies on the optimal-geometry line at 30 °C, with a photon cost (at 30 °C) of 15.5 photons per CO_2_. The first point lies within the observed range of *Bienertia* cell dimensions; the second is positioned slightly outside this range. The CO_2_ leakage is zero at 10°C because both points lie within the C_3_ region of the photon cost landscape at that temperature. Parameters are as in Supplementary Table S1.

Figure 7a–c shows how the photon cost landscape changes with temperature. The cost rises with temperature, and the photon cost valley shifts to smaller core radii; this can be explained by the combination of a lower Rubisco specificity and a higher ratio of dissolved O_2_ to CO_2_ at a higher temperature, which makes it necessary to concentrate the CO_2_ intake from the cell surface into a smaller core volume. The photon cost also rises with temperature in the C_3_ region of the landscape, becoming equal to or larger than its value in the C_4_ valley region. C_4_ photosynthesis thus becomes a winning strategy across the landscape at higher temperatures.

To ascertain the temperature response of an individual plant, in Fig. 7d, e we make a comparison of photosynthetic efficacy measures at a fixed cell geometry (assuming that a cell can ‘throttle’ its C_4_ pump, by adjusting its PEPC and NAD-ME levels, in response to a temperature change, so as to optimize its photosynthetic efficiency at that temperature). The comparison is made at two points in the photon cost landscape, each lying on an optimal-geometry line at a particular temperature (20 °C in Fig. 7d and 30 °C in Fig. 7e). The photon cost at a fixed cell geometry grows steadily with temperature, but the CO_2_ leakage, as well as the carbon assimilation rate (see Discussion), is maximal at the temperature at which the particular cell geometry lies on the optimal-geometry line. An increase in temperature, beyond the value at which a plant’s cell geometry is optimal, results in increased Rubisco activity leading to more RuBP carboxylation (and thus to lower CO_2_ leakage), but also to more RuBP oxygenation (and thus more photorespiration), which lowers the net carbon assimilation rate and increases the photon cost. The CO_2_ leakage, which is often used as a proxy for estimating the efficacy of C_4_ photosynthesis (lower leakage translating to better performance), is thus in fact maximized under optimal photosynthesis conditions. A reduction in CO_2_ leakage with an increase in temperature has been reported in multiple experiments (Kubien *et al.*, 2003; Stutz *et al.*, 2014; von Caemmerer *et al.*, 2014). Our model suggests that this reduction may be a consequence of an adaptive re-optimization of C_4_ biochemistry in plants that optimally photosynthesize at a lower temperature.

#### Varying the permeability of the cell boundary

**Fig. 8.**
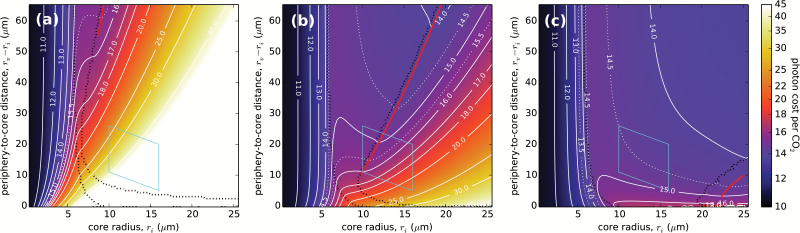
C_4_-optimized photon cost landscapes for three values of the cell boundary permeability. (a) σ_B_=10 μm s^–1^; (b) σ_B_=10^2^ μm s^–1^ (as Fig. 3); (c) σ_B_=10^3^ μm s^–1^. Lines are as in Fig. 3. Other parameters are as in Fig. 2.

The optimal-geometry line and the photon cost valley in Fig. 7 move entirely out of the observed range of central compartment and cell sizes at temperatures beyond 30 °C, seemingly bringing into question the model’s accuracy or the assumption that the photon cost is the major selective pressure. However, the photon cost can also vary significantly with the permeability of the cell boundary. In Fig. 8 the combined cell wall and membrane permeability, σ_B_, is varied across two orders of magnitude, covering the range of estimates in the literature (Terashima *et al.*, 2006; Evans *et al.*, 2009). (Note also that the occlusion of internal airspace by other mesophyll cells translates to a lower effective permeability of the cell barrier.) Lowering σ_B_ to 10 µm s^–1^ leads to a large shift of the C_4_ region in the photon cost landscape towards larger periphery to core distances (i.e. thicker vacuoles), placing the predicted size of a C_4_ pathway-utilizing organism completely outside the observed range (Fig. 8a). The reason for this shift is that the CO_2_ concentration in the centre (Supplementary Fig. S4) is significantly reduced due to the lower CO_2_ intake at the cell surface, which is limited by σ_B_. Achieving the necessary level of CO_2_ concentration to quench photorespiration properly—and so make the C_4_ pump profitable—now requires a larger surface area, hence a larger cell. Increasing the permeability to 1000 µm s^–1^, on the other hand, leads to a shift of the abundant-PEPC region toward larger core radii (Fig. 8c). This shift is due to the higher maximal CO_2_ intake at the cell’s surface, which allows for efficient photorespiration quenching even when the C_4_ pump is not running at full capacity, expanding the region of the finite-PEPC regime.

The position of the photon cost valley and the optimal-geometry line in these two extreme cases is far from the observed range of the central compartment and cell sizes for *Bienertia*. The effective permeability of the cell boundary (taking the occlusion by other mesophyll cells into account) can thus be no lower than ~10^2^ µm s^–1^, as that would lead to an exorbitantly high photon cost. Increasing the permeability substantially beyond 10^2^ µm s^–1^ also takes the optimal-geometry line outside the observed size range (Fig. 8c), but we cannot conclusively reject a high (10^3^ µm s^–1^) permeability value, since the effect mingles with the effects of temperature variation: at 40 °C the high-end permeability estimate places the optimal-geometry line back into the observed sizes range (Supplementary Fig. S5). Because of the conjectural nature of our temperature dependency forecasts, this cannot serve as a definite indicator of the actual value of the cell boundary permeability, but it is clear from Fig. 8 and Supplementary Fig. S5 that increasing σ_B_ results in an efficiency boost in the case of single-cell C_4_ photosynthesis.

## Discussion

We have developed a spatial model of single-cell C_4_ photosynthesis in a cellular geometry typical of *Bienertia* mesophyll cells. It includes the key enzymes of the C_4_ pathway (the primary carboxylase PEPC, and the final decarboxylase NAD-ME), Rubisco carboxylation and oxygenation kinetics, a streamlined photorespiratory cycle, and O_2_ production via the Hill process. The model allowed us to quantify the efficacy of C_4_ photosynthesis and to examine the influence of various factors on the efficiency of the C_4_ pump. We demonstrated that the *Bienertia* mesophyll cell geometry allows for a functional and frugal C_4_ photosynthetic pathway provided the cell is sufficiently large. It can then accommodate a sizeable core compartment with sufficient separation from the peripheral cytoplasm. There is an optimal core size for a given cell size, that minimizes the carbon fixation cost. A C_4_ pump in cells with a thus optimized geometry is an evolutionarily stable advantage against small perturbations in cell dimensions since the photon cost landscape has a valley. The pathway’s efficiency, expressed in terms of the photon cost of carbon fixation, rises with a further increase in cell size, although for cells larger than ~50 µm in radius further gains appear to be marginal.

A photon cost of ~14–16 photons per CO_2_ is achievable for this C_4_ system (Fig. 3), with the CO_2_ leakage ranging between 20% and 35% (Fig. 6b). This can be achieved for reasonably sized cells—the photon cost drops to 15 photons per CO_2_ for cells that are 38 µm in radius, with a 12 µm radius core (Fig. 3). These costs are similar to the lowest costs measured in a number of C_4_ species, where photon costs under limiting light were estimated to be 13–17 photons absorbed per assimilated CO_2_ or evolved O_2_ (Ehleringer and Björkman, 1977; Furbank *et al.*, 1990; Lal and Edwards, 1995). Our model can thus explain how *Bienertia*’s C_4_ system achieves an efficacy comparable with that of other C_4_ plants.

We have shown that the diffusive resistance due to spatial separation can, alone, reproduce the observed levels of CO_2_ leakage from the core compartment in *Bienertia* (von Caemmerer *et al.*, 2014). The leakage in the model does not exceed 36%, a value comparable with those measured in Kranz-type C_4_ plants (Kubásek *et al.*, 2007). Our examination of temperature dependence suggests that, assuming a plant can adjust the levels of the C_4_ cycle enzymes, CO_2_ leakage will be maximal at the temperature at which its cellular/leaf anatomy is best adapted for photosynthesis. At higher temperatures, the leakage decreases—a tendency observed in experiments on C_4_ plants (Kubien *et al.*, 2003; Stutz *et al.*, 2014; von Caemmerer *et al.*, 2014). The increase in carbon assimilation rate, combined with the effective quenching of Rubisco oxygenation activity via carbon-concentrating mechanisms, would provide a vast advantage to C_4_ pump-utilizing plants at high temperatures. In contrast, at low temperatures a plant would benefit from throttling down the C_4_ pump or shutting it down completely. In this regard, we note that while the carbon isotope composition analysis of leaf biomass in *Bienertia* that grew in natural habitats in Central Asia (under high light and warm climate conditions) consistently indicate a C_4_-type carbon isotope composition (Akhani *et al.*, 2005), the experiments with chamber-grown *Bienertia* plants suggest that environmental conditions can influence the expression of the C_4_ pump, the carbon isotope composition ranging from C_4_ to C_3_–C_4_ intermediate values (Stutz *et al.*, 2014).

Based on the energy cost of carbon fixation, the local selection pressure within the photon cost valley would keep the C_4_ pump operational and select for increasing the size of the cell and of the core region so as to reach and then slide along the optimal-geometry line. On shorter time scales, optimization pressures could also guide a maturing *Bienertia* mesophyll cell to follow the same path. The valley in the photon cost landscape only appears at cell radii larger than ~25 µm, so a smaller cell would prefer to keep its plastids undifferentiated, utilizing the C_3_ photosynthetic pathway. Photon cost optimization thus explains why activation of the C_4_ pump in *Bienertia* cells occurs only once they reach a certain size [as witnessed in specialization of plastid biochemistry (Voznesenskaya *et al.*, 2005; Park *et al.*, 2009)], but it cannot explain how this cellular architecture evolved in the first place, as there is no continuous evolutionary path of decreasing cost from the C_3_ to the C_4_ region in the photon cost landscape. A path of low resistance goes over a ‘hill’ in the landscape at *r*_i_ ~6 µm. The climb is not steep—the photon cost rises from 12 at *r*_i_ ~3 µm to <15 at the summit—but this ‘crossing’ is possible only at a large enough *r*_v_–*r*_i_, in other words when a cell is already large. Hence, other factors had to contribute to the initial increase in cell size before the improvement of photosynthetic efficacy led to the development of a C_4_ pathway. It has been suggested that the evolution of the C_4_ mechanism occurred via intermediates, where the first step in increasing photosynthesis under limiting CO_2_ was to develop two domains, with photorespired CO_2_ refixed in the internal domain (‘C_2_ photosynthesis’) (Sage *et al.*, 2012).

**Fig. 9.**
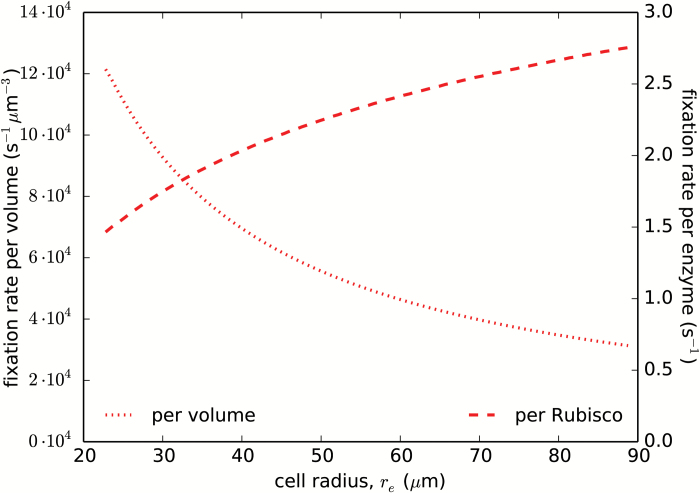
Net carbon assimilation rates per cell volume and per Rubisco enzyme. The assimilation rates are compared along the optimal-geometry line (the red line in Fig. 3). Parameters are as in Fig. 2.

Secondary factors that can be important determinants of a plant’s fitness are the specific carbon assimilation rates, notably the net per-Rubisco assimilation rate and the net assimilation rate per cell volume. The importance of the per-Rubisco assimilation rate stems from the high cost of Rubisco production, which tends to consume a major share of the cell’s resources (it is the most abundant protein in the cell). The per-volume assimilation rate is also important since general cellular maintenance costs grow with cell volume. The two assimilation rates are shown along the optimal-geometry line in Fig. 9. The region of high per-Rubisco assimilation rate matches the location of the photon cost valley (Supplementary Fig. S6a), and the assimilation rate rises along the optimal-geometry line by 40% as the cell’s radius increases from 30 µm to 70 µm. In contrast, the per-volume assimilation rate drops sharply with cell size (Supplementary Fig. S6b). This places an upper limit on the cell size, as the net carbon assimilation rate would have to cover the daily respiratory losses as well as growth demands, which increase with cell size.

The per-volume assimilation rate is not the only factor that will constrain the maximal cell size. The PEPC requirements in larger cells are another, as the model generally predicts a high PEPC concentration in the C_4_ valley region. This is consistent with the measured ratio of total PEPC and Rubisco carboxylation capacities in *Bienertia* mesophyll cells, which is ~5 (von Caemmerer *et al.*, 2014), corresponding to the abundant-PEPC regime in our model. The third limiting factor, which is implicit in the model, is the diffusive transport of C_3_ and C_4_ acids between the core and the periphery. This transport has to support the carboxylation current, but is likely to be constrained to narrow cytoplasmic strands penetrating the vacuole (Voznesenskaya *et al.*, 2005), so it may be rate limiting. The fraction of vacuole area covered by the strands is unknown. If a conservative estimate of 1% is assumed, the concentration of C_3_ acids in the core and C_4_ acids in the periphery would have to be >100 mM to sustain the predicted carboxylation flux in very large cells (*r*_e_ >70 µm) (Supplementary Fig. S7). Within the region of observed *Bienertia* cell sizes, however, the required difference in C_3_ and C_4_ acid concentrations between the core and the periphery regions would be ≤50 mM, which is comparable with concentration differences found in Kranz anatomy C_4_ species (Hatch and Osmond, 1976). Photosynthesis may also be constrained by light levels and cyclic versus linear flow balance (Björkman and Demmig-Adams, 1995; Zhu *et al*., 2008). In particular, the peripheral chloroplasts appear to be PSII deficient (Voznesenskaya *et al.*, 2002), although they seem to retain some capacity for linear electron flow (Offermann *et al.*, 2011). Examination of these concerns (Supplementary Figs S8, S9) shows that, without NADPH production in the periphery, *Bienertia* mesophyll cells would require very high insolation to achieve the optimal assimilation rate. A co-ordinated energy production between the peripheral and core chloroplasts may be necessary to support C_4_ photosynthesis, while light availability could be a limiting factor to carbon assimilation rate and to mesophyll cell size in *Bienertia*.

The main model results are robust to model assumptions and to poorly known parameters. Plasma membrane and cell wall permeability is a key factor in limiting transport of CO_2_, but is difficult to measure. Our model shows that *Bienertia* is not viable with permeabilities near the lower end of their estimated range (10 µm s^–1^), while permeabilities in the mid range of 100 µm s^–1^ give realistic photon costs at 20 °C, with an optimal geometry close to that observed (Fig. 8). At a higher temperature (~40 °C) those conditions are again satisfied, if a permeability close to the higher end of the estimated range (10^3^ µm s^–1^) is assumed. The pronounced sensitivity of the photon cost to some of the parameters, notably the cell boundary permeability and the temperature dependence of Rubisco kinetics, shows that more reliable measurements are needed in order to model photosynthesis accurately.

The model assumes a uniform mixture of chloroplasts and mitochondria in the central compartment. The micrographs of *Bienertia* cells, however, suggest that mitochondria in the CCC are positioned closer to its centre, surrounded by chloroplasts (Voznesenskaya *et al.*, 2002; Park *et al.*, 2009; Lung *et al.*, 2011). To see the impact of mitochondrial positioning, we explored what happens when the mitochondrial release of CO_2_ is limited to the inner part of the core. Supplementary Fig. S10 shows that the impact is marginal—resulting in approximately half a photon decrease in photon cost—even in an extreme case when the mitochondrial release is constrained to the central 11% of the core region volume. There is little change to the assimilation rate, even though the central CO_2_ concentration increases by an order of magnitude (Supplementary Fig. S10c). The CO_2_ leakage is, however, substantially reduced, dropping from 36% to 28% at the start of the optimal-geometry line (Supplementary Fig. S10d). The large drop in CO_2_ leakage results only in miniscule gains in the photon cost along the optimal-geometry line because photorespiration is already fully suppressed.

Changing the type of Rubisco expressed in the core, from a C_4_ variety (maize; Cousins *et al.*, 2010) to C_3_ varieties [such as from spinach (Zhu *et al.*, 1998) or wheat (Cousins *et al.*, 2010)], leads to a minor increase in photon cost of up to 1 photon per CO_2_, but does not noticeably change the position or the depth of the C_4_ valley (Supplementary Fig. S11). Changing the concentration of Rubisco in the core moves the position of the optimal-geometry line but does not alter the photon cost at its starting point (Supplementary Fig. S12). The per-volume assimilation rate increases with Rubisco concentration (Supplementary Fig. S12d). The chloroplasts, however, tend to be already fully packed with Rubisco, and mixing them with mitochondria in the core can only lower the effective Rubisco concentration. The cell probably has to establish a fine balance between filling the core with chloroplasts and providing sufficient mitochondria to decarboxylate the incoming malate. Gathering the mitochondria in the inner part of the core, which had little direct impact on photosynthetic efficiency or assimilation rates, may be advantageous in this regard. Another scenario where it might prove beneficial is when, for whatever reason, the C_4_ pump runs suboptimally and photorespiration is high. The reduced CO_2_ leakage due to mitochondrial central localization would then translate into more tangible improvements in the photon cost.

Reducing the CO_2_ concentration in the surrounding airspace (Supplementary Fig. S13) increases the cost of carbon fixation, but also makes the C_4_ valley deeper, showing that the C_4_ pump provides a greater advantage under conditions of CO_2_ deprivation (such as when stomata are closed). Even changing the external environment from air to water, with the corresponding 10 000-fold decrease in the diffusion rate outside the cell (Lugg, 1968; Mazarei and Sandall, 1980), results in little qualitative change in the photon cost landscape (Supplementary Fig. S14). This suggests that a *Bienertia*-like cell architecture could also provide an evolutionary advantage to aquatic plants. The cells would need to be somewhat larger—the photon cost valley starts at a periphery to core separation of 23 µm. This value is comparable with the general size ranges of single-cell C_4_ aquatic plants *Hydrilla verticillata* and *Orcuttia viscida* (Keeley, 1998; Bowes *et al.*, 2002; von Caemmerer *et al.*, 2014) (though they have a different cell geometry). The same criterion would, however, disqualify the allegedly C4-photosynthesizing diatom *Thalassiosira weissflogii* (Reinfelder *et al.*, 2000, 2004).

The prospect of introducing a C_4_ pathway into C_3_ crop plants has been investigated in recent years, with the aim of enhancing photosynthesis and productivity (von Caemmerer, 2003; Zhu *et al.*, 2010). An approach based on a single-cell C_4_ cycle is appealing as it circumvents the need to engineer Kranz anatomy into the plant. Our results show that such approaches hold promise, provided that the mesophyll cells are sufficiently large, or can be engineered to be sufficiently large. Targeting PEP carboxylation and malate decarboxylation to specific locations within the cell could then create the required spatial separation. Surprisingly, this separation need not be very large: in our model, the photon cost drops below 15 at periphery to core separations of just over 21 µm—even without imposing any diffusion barriers between the carboxylation and decarboxylation regions. On the other hand, where an adequate spatial separation is not feasible, such as on the level of individual chloroplasts, engineering a C_4_ pump would not provide any gains.

## Supplementary data

Supplementary data are available at *JXB* online

Supplementary model description

Table S1. Parameter values for modelling C_4_ photosynthesis at different temperatures.

Figure S1. Optimal PEPC and NAD-ME concentrations.

Figure S2. Oxygen concentration in the cell centre.

Figure S3. Optimized photon cost along the optimal-geometry line.

Figure S4. CO_2_ concentration in the cell centre at different cell boundary permeabilities.

Figure S5. Photon cost landscape at 40 °C with the cell boundary permeability of 10^3^ µm s^–1^.

Figure S6. Net carbon assimilation rates.

Figure S7. C_4_ acid concentration levels needed to drive the C_4_ pump.

Figure S8. Fraction of the electron current due to cyclic electron flow.

Figure S9. Photosynthetically active photon flux needed to sustain photosynthesis.

Figure S10. Repositioning mitochondria at the centre of the core region.

Figure S11. Comparison of C_4_ pump efficiency for differing Rubisco enzyme characteristics.

Figure S12. Varying the Rubisco concentration in the core.

Figure S13. Varying the ambient CO_2_ concentration.

Figure S14. Comparison of water and air environments.

## Supplementary Material

Supplementary_Model_S1

Supplementary_Table_S1_Figures_S1_S14
