# Supplementary material for: Size matters for single-cell C4 photosynthesis in Bienertia
Source: J Exp Bot. 2016 Oct 12;68(2):255–67. doi: 10.1093/jxb/erw374 (PMC5853773; doi:10.1093/jxb/erw374)
Supplement: Supplementary_Model_S1 [file erw374_suppl_supplementary_model_s1.pdf]

Size matters for single-cell C_4 photosynthesis in *Bienertia*: Supplementary material (the model)

Ivan Jurić[a,b], Vinicio González-Pérez[a], Julian M. Hibberd[c], Gerald Edwards[d], Nigel J. Burroughs[a]

[a] *Warwick Systems Biology Centre, University of Warwick, Coventry, CV4 7AL, UK*
[b] *Institute of Physics, Bijenička c. 46, P.O. Box 304, HR-10001 Zagreb, Croatia*
[c] *Department of Plant Sciences, University of Cambridge, Cambridge, CB2 3EA, UK*
[d] *School of Biological Sciences, Washington State University, Pullman, WA 99164-4236, USA*

S.1. Extended model justification and overview

S.1.1. Elements of the model

Our model considers a single spherical *Bienertia* mesophyll cell, divided into three concentric compartments. In the centre, we have the CCC composed of mitochondria and Rubisco-rich chloroplasts. It is surrounded by one large concentric vacuole interspersed with cytoplasmic channels that connect the central region with the periphery. We do not model these channels explicitly, but merge them with the vacuole interior into an effective medium through which gases can diffuse. The peripheral region, contains chloroplasts rich in enzymes that fix inorganic carbon into aspartate as part of the C_4 photosynthetic pathway. We refer to the three compartments as "the core", "the vacuole", and "the periphery". We note that *Bienertia* mesophyll cells are not actually spherical. However, since our aim is to look at the general effects of size on the efficiency of a C_4 pathway, a simpler model (which is also more amenable to numerical investigation) will suffice. Within this geometry, we consider the diffusion of carbon dioxide and oxygen in the cell, as well as the processes of fixation of carbon into C_4 acids in the periphery and its subsequent release in the core, and the processes of carboxylation and oxygenation of RuBP by Rubisco. The latter starts a complex chain of photorespiratory reactions, resulting in a loss of carbon from the cell's sugar store, which is released as CO_2 in the mitochondria in the CCC. We also consider oxygen production by photosystem-II in the CCC chloroplasts, which is associated with the production of NADPH that is needed for RuBP regeneration and photorespiration.

It is important to emphasise that, although we conceptually divide the cell's interior into distinct spatial regions, the model places no *intracellular* barriers that would hinder diffusion of oxygen and CO_2 between these regions. This is a deliberate choice, to test the viability of the C_4 pump when there is nothing but spatial separation to provide diffusive resistance to gases in the liquid phase. It is clear that placing additional barriers would further improve the efficiency of the C_4 photosynthesis by decreasing the leakage of CO_2 from the core, provided the transport of substrates and C_4 acids is unhindered.

Since the details of the C_4 pathway vary among plant species (von Caemmerer and Furbank, 2003; Jenkins et al., 1989; Sage, 2004), we abstract the pathway into its two essential steps. First is the fixation of atmospheric CO_2 into a C_4 acid by PEPC in the periphery. The second is the decarboxylation of the C_4 acid by NAD-ME in the

core region, which frees the captured carbon. By ignoring the remaining steps in the C_4 cycle we implicitly assume they are not rate limiting. This, for instance, implies that the base C_3 substrate (alanine) as well as the C_4 product (aspartate) are abundant within the cell - a necessary condition for optimal functioning of the C_4 pathway in any case.[1] Similar assumptions are placed on all the steps of the C_3 pathway which involve Rubisco, prior to carboxylation or oxygenation of RuBP (i.e. Rubisco activation, RuBP binding, etc.), since carbon-fixation is the limiting step there.

Our model thus explicitly considers three enzymes: Rubisco (active site concentration c_R in the CCC), PEPC (active site concentration c_P in the periphery), and NAD-ME (parametrised by a CO_2 mitochondrial current Ψ_{mitoC} in the CCC). The model also explicitly considers two mobile inorganic molecular species - carbon dioxide and oxygen - whose concentrations in steady-state, $c_C(r)$ and $c_O(r)$, will vary with distance, r, from the cell centre. The enzymatic reactions of CO_2 and oxygen follow Michaelis-Menten kinetics. Since detailed kinetics data for *Bienertia*'s Rubisco are not presently available, we use kinetic parameters for maize (*Zea mays*) (Cousins et al., 2010), a well-studied C_4 plant (the carboxylation catalytic rates, k_{catC}, for maize and *Bienertia* Rubisco are similar, and higher than in C_3 plants (Rosnow et al., 2015)). PEPC and NAD-ME kinetic parameters are taken for *Zea mays* and *Arabidopsis thaliana* respectively (Kai et al., 1999; Tronconi et al., 2008). Values of all the physical and chemical parameters used in the simulations are listed in Table 1. In addition to these explicitly treated enzymatic processes, we also take into account the release of carbon dioxide in mitochondria (photorespiration) and oxygen in the core chloroplasts (Hill reaction at PS-II). The photorespiratory CO_2 release is set to match half the Rubisco's oxygenation current, while the Hill reaction is set to produce oxygen by water cleavage by the amount needed to replenish the NADPH lost running the Calvin-Benson and the photorespiratory cycle (this amounts to one oxygen molecule produced for every RuBP carboxylation or oxygenation event).

The efficacy of the photosynthetic pathway will be determined by three principal factors, firstly the C_4 pump reaction kinetics (PEPC concentration, NAD-ME concentration), secondly the Rubisco concentration, and thirdly the cell geometry, i.e. the radii of the three compartments. To analyse the effects of the geometry, we optimise the C_4 pump reaction kinetics, i.e. for a given choice of geometry and the core Rubisco concentration, we tweak the biochemistry of the C_4 pump by varying the concentration of PEPC in the periphery to optimize its performance during steady state carbon fixation, see S.2. The concentration of NAD-ME in the core is automatically adjusted, for any given PEPC concentration, so as to balance the PEP-carboxylation in the periphery with the malate-decarboxylation in the core.[2] This implies a regulatory mechanism for NAD-ME expression, based on the concentration of C_4 acids (aspartate and/or malate) within the central compartment.

S.1.2. CO_2 and HCO_3^-

We do not explicitly model bicarbonate in the cell. This is because it effectively decouples from CO_2 except in the periphery, where the presence of carbonic-anhydrase (CA) ensures rapid equilibration. Elsewhere, the absence of

[1] It also means that the ATP consumption connected to pyruvate-to-PEP conversion via PPDK enzyme is now effectively associated with the carbon capture step which it precedes.

[2] In the limit of high malate concentration ($\gtrsim 100K_M \approx 30\,\text{mM}$) that we are interested in, NAD-ME will be saturated by malate, and the malate-decarboxylation current will be directly proportional to NAD-ME concentration.

CA means the interconversion between CO_2 and HCO_3^- will be slow - much slower than other (diffusive and kinetic) processes. In the periphery, HCO_3^- will be at equilibrium with local CO_2 concentration, as CA-assisted $CO_2 \leftrightarrow HCO_3^-$ interconversion is much faster than PEPC carboxylation (Heinhorst et al., 2006). Bicarbonate can then be taken 'out of the equation', as it only affects the effective PEPC Michalis-Menten constant for CO_2, $K_{P(CO_2)} = \frac{c_{CO2}}{c_{HCO3-}} K_P(HCO_3^-) \approx \frac{1}{20} K_P(HCO_3^-)$ at pH = 7.5.[3]

This approximation is valid if $CO_2 \leftrightarrow HCO_3^-$ interconversion can be neglected in the core and vacuole regions. To see this is indeed the case, let us consider the fate of a CO_2 molecule released from the core mitochondria (either as a photorespiration product or from malate-to-pyruvate conversion). Without CA it converts to HCO_3^- slowly (Johnson, 1982) - much slower than the time it takes for it to diffuse out of the core region or to react with Rubisco. The relevant lengthscale will be the average CO_2 diffusion distance, $\lambda = \sqrt{D_C/k_{CO_2}}$, where k_{CO_2} is the overall $CO_2 \rightarrow HCO_3^-$ conversion rate. The rate depends on pH, but will generally be below $7 \cdot 10^{-2}\,\mathrm{s}^{-1}$ for pH≤8.5 (Johnson, 1982), giving $\lambda \geq 160\,\mu\mathrm{m}$. This is well above the largest Bienertia cell radius considered in the paper. Once in the periphery, the conversion becomes rapid, thanks to CA. The fate of HCO_3^- molecules in the cytoplasm outside the periphery will be similar. The back-reaction $HCO_3^- \rightarrow CO_2$ is roughly twenty times slower than the forward reaction, while diffusion constants of CO_2 and HCO_3- are comparable (Mazarei and Sandall, 1980; Walker et al., 1980), so any HCO_3^- molecule will most likely end up captured by PEPC.

Note that, while the model assumes no CA in the core region, the conclusions would remain the same if CA were present in the stroma of the core plastids. This is because, even though CA would cause rapid equilibration of CO_2 and HCO_3^-, the HCO_3^- within the stroma is effectively trapped (it is confined to individual chloroplasts) and it does not react with any other enzymes in the model. At steady-state, the HCO_3^- concentration within the stroma would thus simply be proportional to the CO_2 concentration.

To improve the treatment of CO_2/HCO_3^- kinetics, one would also need to consider (and have the experimental knowledge of) the variation in pH and the CA distribution amongst the different compartments, as well as explicit modelling of transport through cytoplasmic channels. This would greatly increase the complexity of the model, and the uncertainties in the numerous newly introduced parameters would have to be addressed.

S.1.3. Determination of the photon cost

There are several efficacy measures that can be used to evaluate the net carbon fixation. We use the photon cost of carbon fixation (the inverse of the base quantum yield), which we refer to simply as 'the photon cost'. This is the minimal number of photons that, on average, need to be collected by the linear and cyclic photosystems to regenerate the ATP and NADPH used in the process of net fixation of one carbon atom into sugar.[4] It covers the cost of RuBP regeneration, the photorespiratory cycle, and the C_4 pump operation. If the optimal cost is achieved at non-vanishing

[3] This results in a rescaling of the effective PEPC-CO_2 reaction rate, $k_{onP} = 20\frac{k_{catP}}{K_P}$)

[4] We do not consider additional costs - for example, due to the inefficiencies in photon collection by the photosystem's antenna complex. These considerations fall outside the scope of this paper.

PEPC and NAD-ME concentrations (i.e. when the C_4 pump is active), we can say that the C_4 photosynthetic pathway is a viable and preferable alternative to C_3-only photosynthesis for the selected cell geometry.

The net photon cost can be expressed as

$$\varphi = \frac{\varphi_C \Phi_C + \varphi_O \Phi_O + \varphi_{C4} \Phi_{C4}}{\Phi_C - \Phi_O/2} \tag{S1}$$

where φ_C, φ_O, and φ_{C4} are base costs of RuBP regeneration after one Rubisco-carboxylation event, of the photorespiratory salvage cycle after an oxygenation event, and of one pyruvate-to-PEP conversion (which has to occur for each PEP-carboxylation event in the steady-state). These values are estimated at $\varphi_C = 8$, $\varphi_O = 9$, and $\varphi_{C4} = 4$ (Zhu et al., 2010). Φ_C, Φ_O, and Φ_{C4} are the total RuBP-carboxylation current, RuBP-oxygenation current and the C_4 current.

These values correspond to optimal utilisation of the linear and cyclic electron transfer chains in producing the required amounts of ATP and NADPH to run the above processes. The amounts are 3 ATP and 2 NADPH for each carbon atom assimilated via Calvin-Benson cycle, 3.5 ATP and 2 NADPH for the photorespiratory cycle after each oxygenation event, and 2 ATP for every pyruvate-to-PEP conversion (Farquhar et al., 1980; Zhu et al., 2010; Kramer and Evans, 2011). The ATP and NADPH requirements translate into photon cost as follows. One turn of the linear electron transfer chain involves absorption of 4 photons (2 by photosystem I and 2 by photosystem II), which are used to tranfer 6 protons into the thylakoid lumen and reduce one $NADP^+$ molecule (Zhu et al., 2010; Kramer and Evans, 2011). ATP synthase uses the resulting proton concentration gradient to phosphorylate ADP. Production of one ATP molecule requires a down-gradient transfer of 4 protons (Zhu et al., 2010).[5] The cyclic electron transfer chain only maintains the proton gradient, so it can be used to produce additional ATP (above the ATP/NADPH ratio of 3/2). The commonly accepted scenario involves transfer of 4 protons for every 2 photons absorbed by photosystem I (Zhu et al., 2010).[6] Combining the ATP/NADPH requirements of photosynthetic processes with the productivities of the transfer chains, we obtain the stated photon cost values.

S.1.4. External environment

We consider the cell placed in one of two possible environments. One is a gas phase (air), the other is water. In the case of a gaseous environment, we place a diffusion barrier, in the form of the plasma membrane and cell wall, that hinders the exchange of CO_2 and oxygen between the cell and the gas phase. By varying the permeability of this barrier we can also effectively account for partial occlusion of our cell by its neighbours. We make the standard assumption that the gas solvation at the cell boundary is a fast process (Tholen and Zhu, 2011), so that the concentrations of CO_2 and O_2 in a thin hydrated layer immediately beyond the cell wall are in equilibrium with their partial pressures

[5]The exact efficiency of ATP synthase is a matter of active research. There are indications that production of an ATP molecule requires more than 4 protons on average, and that the actual efficiency varies among species (Kramer and Evans, 2011). Four protons per ATP is a commonly used value when estimating the efficiency of photosynthesis (Zhu et al., 2010).

[6]There are indications that the cyclic electron transfer chain is in fact twice as efficient as commonly assumed (Kramer and Evans, 2011). If so, the photon cost of processes which involve only ATP consumption - such as the C_4 pump operation - would be halved. As our general goal is to provide a conservative estimate of the single-cell C_4 photosynthetic pathway efficiency, we assume the lower productivity (of 2 protons per photon) for the cyclic chain.

in air. In the second scenario, the cell is immersed in water. By considering the functioning of the C_4 pump in an aqueous environment, where diffusion is slow to the point of limiting the CO_2 flux, we can assess the efficacy of carbon-concentrating mechanisms in relation to cell size in single-cell C_4 aquatic plants (von Caemmerer et al., 2014; Reinfelder et al., 2000). The external concentrations of dissolved CO_2 and O_2 are, in this case, set to be at equilibrium with their partial pressures in air at a large distance from the cell, and will notably deviate from equilibrium in the cell's proximity.

S.2. Equations of the model

We model the *Bienertia* mesophyll cell as a spherically symmetric system of three concentric compartments and an exterior. The inner compartment ("the core"), is a sphere of radius r_i. It is surrounded by vacuole mantle, up to radius r_v from the centre. The vacuole is followed by a thin shell ("the periphery") up to the external radius of the cell, r_e. Beyond, we have "the outside", which can be either air or water.

S.2.1. Within the cell

We treat the core as an homogeneous mixture of mitochondria and chloroplasts. Rubisco is spread evenly throughout the core, with concentration c_R. It reacts with CO_2 and oxygen, following a Michaelis-Menten type kinetics, with bimolecular reaction rates k_{onC} and k_{onO}, and saturating concentrations K_C and K_O, of CO_2 and O_2 respectively (for parameter values see Table 1). The mitochondria provide a spatially uniform release of CO_2 stemming from photorespiration and malate decarboxylation. The chloroplasts likewise produce a uniform release of oxygen equal to NADPH consumption by carbon fixation and photorespiration. These release rates per unit volume, ψ_{mitoC} and ψ_{chlorO}, are, in steady-state, determined self-consistently by current-balance conditions (Eq. S23 and S24).

To derive the steady-state equations for carbon dioxide and oxygen distributions, $c_C(r)$ and $c_O(r)$, we start from a general set of time-dependent diffusion-reaction equations for the core region ($r < r_i$),

$$\frac{\partial c_C}{\partial t} = D_C \nabla^2 c_C - k_{onC} c_C \left(c_R - c_{RC} - c_{RO}\right) + \psi_{mitoC} \tag{S2}$$

$$\frac{\partial c_O}{\partial t} = D_C \nabla^2 c_O - k_{onO} c_O \left(c_R - c_{RC} - c_{RO}\right) + \psi_{chlorO} \tag{S3}$$

$$\frac{\partial c_{RC}}{\partial t} = k_{onC} c_C \left(c_R - c_{RC} - c_{RO}\right) - k_{catC} c_{RC} \tag{S4}$$

$$\frac{\partial c_{RO}}{\partial t} = k_{onO} c_O \left(c_R - c_{RC} - c_{RO}\right) - k_{catC} c_{RO} \tag{S5}$$

where c_{RC} and c_{RO} are concentrations of Rubisco-RuBP-CO_2 and Rubisco-RuBP-O_2 complexes, and $k_{catC} = K_C k_{onC}$ and $k_{catO} = K_O k_{onO}$ are Rubisco carboxylation and oxygenation catalysis rates. By setting all the time derivatives to zero and simplifying the Laplace operator for the case of a spherically symmetric system, we get the steady-state equations for the radially varying concentrations of CO_2 and oxygen, c_C and c_O, within the core region, $r < r_i$,

$$D_C \frac{1}{r} \frac{\mathrm{d}^2}{\mathrm{d}r^2} \left(r c_C\right) = k_{onC} c_R K_C \frac{c_C K_O}{K_C K_O + c_C K_O + K_C c_O} - \psi_{mitoC} \tag{S6}$$

$$D_O \frac{1}{r} \frac{\mathrm{d}^2}{\mathrm{d}r^2} (r c_O) = k_{onO} c_R K_O \frac{K_C c_O}{K_C K_O + c_C K_O + K_C c_O} - \psi_{chlorO} \tag{S7}$$

Within the vacuole no reactions take place, so the concentrations of CO_2 and oxygen are affected by diffusion only. In the steady-state, the Laplace equation holds for $r_i < r < r_v$,

$$\frac{1}{r} \frac{\mathrm{d}^2}{\mathrm{d}r^2} (r c_C) = \frac{1}{r} \frac{\mathrm{d}^2}{\mathrm{d}r^2} (r c_O) = 0 \tag{S8}$$

In the periphery, CO_2 indirectly reacts with the PEPC enzyme. We assume abundant PEP supply. We also use a simpler form of the reaction rate, linear in c_C.[7]

Starting from the time-dependent equations,

$$\frac{\partial c_C}{\partial t} = D_C \nabla^2 c_C - k_{onP} c_C c_P \tag{S9}$$

$$\frac{\partial c_O}{\partial t} = D_C \nabla^2 c_O \tag{S10}$$

we straightforwardly obtain the steady-state equations valid for $r_v < r < r_e$,

$$D_C \frac{1}{r} \frac{\mathrm{d}^2}{\mathrm{d}r^2} (r c_C) = k_{onP} c_P c_C \tag{S11}$$

$$D_O \frac{1}{r} \frac{\mathrm{d}^2}{\mathrm{d}r^2} (r c_O) = 0 \tag{S12}$$

Solutions to the differential equations for c_C and c_O have to match smoothly at the boundaries between the intracellular regions, i.e. we have conditions $c_C (r \to r_{i-}) = c_C (r \to r_{i+})$ and $\frac{\mathrm{d}c_C}{\mathrm{d}r}\Big|_{r \to r_{i-}} = \frac{\mathrm{d}c_C}{\mathrm{d}r}\Big|_{r \to r_{i+}}$, with analoguous conditions at the $r = r_v$ boundary. To fully determine c_C and c_O, we also need to set the boundary conditions at the centre, $r = 0$, and the cell boundary, $r = r_e$. The first is simply the smoothness requirement at $r = 0$,[8]

$$\left.\frac{\mathrm{d}c_C}{\mathrm{d}r}\right|_{r \to 0} = \left.\frac{\mathrm{d}c_O}{\mathrm{d}r}\right|_{r \to 0} = 0. \tag{S13}$$

The situation at the $r = r_e$ boundary depends on our choice of external conditions.

[7]The use of a linear form permits an analytical solution of the resulting diff. equations, which is handy since the numerical integration of the equations becomes unstable when (as is usually the case) the CO_2 concentration in the periphery is low or vanishing. Where this is not the case, the PEPC concentration at which the optimal solution is found will be underestimated, but the optimal solution itself (in particular the optimal photon cost) should not change, since it is determined by flux-balance and cost considerations. A correction to the optimal PEPC concentration can be estimated by equating the total PEP carboxylation currents when linear and M-M reaction rates are used, assuming the same average CO_2 concentration in the periphery (i.e. $c_{P(MM)} c_{Cav} / \left(K_{P(CO_2)} + c_{Cav}\right) = c_{P(lin)} c_{Cav} / K_{P(CO_2)}$). We do not apply this correction in Fig. 2(a) because it is not exact. The correction becomes notable (a rescaling by a factor between 2 and 4) away from the optimal-geometry line, in the region where the optimal PEPC concentration is small (< 0.01 mM).

[8]A finite slope at $r = 0$ would imply the presence of an infinite-strength source or drain at the origin. The quickest way to see this is to note that the concentration gradient (and hence the current density) would be discontinous at the origin. The continuity equation, $\mathrm{div}\vec{j}_c = \partial_t c$, then implies a divergent right hand side. Another way would be to consider the current into a vanishingly small sphere ($r \to 0$) around the origin. The current through the sphere is proportional to $\frac{\mathrm{d}}{\mathrm{d}r} c (r \to 0) r^2$, which should be equal to the source term $\propto S (r \to 0) r^3$, where $S (r \to 0)$ is the source strength at the origin. Hence, $\frac{\mathrm{d}}{\mathrm{d}r} c (r) \propto r S (r)$ as $r \to 0$.

S.2.2. The choice of exterior

In the case of air outside and a diffusion barrier at r_e, we can safely assume a constant gas concentration outside the cell due to the significantly higher diffusion coefficients in a gaseous phase. We make the standard assumption that the concentrations of CO_2 and O_2 in a thin hydrated layer immediately beyond the cell wall are in equilibrium with their partial pressures in air: $c_C\,(r > r_e) = c_{Ceq}$ and $c_O\,(r > r_e) = c_{Oeq}$. In this case the flow (per unit area) of dissolved CO_2 (j_C) and oxygen (j_O) at the cell boundary will be determined by the cell wall and cell membrane permeability, σ_B. This flow must match the diffusive flow within the cell, at the cell membrane

$$j_C = \sigma_B \left(c_{Ceq} - c_C\,(r \to r_{e-}) \right) = D_C\, \left.\frac{\mathrm{d}c_C}{\mathrm{d}r}\right|_{r \to r_{e-}} \tag{S14}$$

$$j_O = \sigma_B \left(c_{Oeq} - c_O\,(r \to r_{e-}) \right) = D_O\, \left.\frac{\mathrm{d}c_O}{\mathrm{d}r}\right|_{r \to r_{e-}} \tag{S15}$$

In the case of a water environment, the concentrations of CO_2 and oxygen outside the cell will noticeably vary with distance (following the Laplace equation, Eq. S8), due to the limitations of diffusive transport. The boundary condition at the infinity requires that c_C and c_O approach their equilibrium dissolved values, c_{Ceq} and c_{Oeq}.[9] Using the Laplace equation outside the cell, we obtain a matching condition at $r = r_e$,[10]

$$r_e\, \left.\frac{\mathrm{d}c_C}{\mathrm{d}r}\right|_{r_e} + c_C(r_e) = c_{Ceq} \tag{S16}$$

$$r_e\, \left.\frac{\mathrm{d}c_O}{\mathrm{d}r}\right|_{r_e} + c_O(r_e) = c_{Oeq} \tag{S17}$$

S.2.3. The currents

The final ingredients are the determination of mitochondrial CO_2 release rate ψ_{mitoC} and the plastid oxygen release rate ψ_{chlorO}. At steady-state, the total mitochondrial CO_2 release current (Φ_{mitoC}) must match the C_4 acid current (Φ_{C4}) and the photorespiratory release current, which is half the Rubisco oxygenation current (Φ_O). The oxygen release current (Φ_{chlorO}) must produce enough NADPH to support both the Rubisco carboxylation (Φ_C) and Rubisco oxygenation current (i.e. both the Calvin-Benson cycle and the photorespiratory requirements for reducing power). Two NADPH molecules are needed per each RuBP carboxylation or oxygenation event, and one NADPH is produced for each H_2O molecule split in the Hill process; hence two are produced for every O_2 evolved.

The currents are given by

$$\Phi_C = \int_0^{r_i} 4\pi r^2 \cdot k_{onC} c_R K_C \frac{c_C K_O}{K_C K_O + c_C K_O + K_C c_O} \mathrm{d}r \tag{S18}$$

$$\Phi_O = \int_0^{r_i} 4\pi r^2 \cdot k_{onO} c_R K_O \frac{K_C c_O}{K_C K_O + c_C K_O + K_C c_O} \mathrm{d}r \tag{S19}$$

[9] We ignore the bicarbonate pool outside the cell because HCO_3^- cannot pass the cell membrane in any significant amount.

[10] Concentrations of oxygen and CO_2 outside follow a simple diffusion law, $D \frac{1}{r} \frac{\mathrm{d}^2}{\mathrm{d}r^2}(rc) = 0$, (i.e. same as Eq. S12). The solution is of the form $rc\,(r) = Ar + B$. By noting that $A = c\,(r \to \infty)$ and $\frac{\mathrm{d}c}{\mathrm{d}r} = -\frac{B}{r^2}$, we can get a general expression, valid for any $r \geq r_e$: $r \frac{\mathrm{d}}{\mathrm{d}r} c\,(r) + c\,(r) = c\,(r \to \infty)$.

$$\Phi_{C4} = \int_{r_v}^{r_e} 4\pi r^2 \cdot k_{onP} c_P c_C \mathrm{d}r \tag{S20}$$

$$\Phi_{mitoC} = \frac{4\pi}{3} r_i^3 \psi_{mitoC} \tag{S21}$$

$$\Phi_{chlorO} = \frac{4\pi}{3} r_i^3 \psi_{chlorO} \tag{S22}$$

The balance conditions can now be stated as

$$\Phi_{mitoC} = \Phi_{C4} + \Phi_O/2 \tag{S23}$$

$$\Phi_{chlorO} = \Phi_C + \Phi_O \tag{S24}$$

The differential equations S6-S8 and S11-S12, along with the boundary (S13 and S16-S17 or S14-S15) and current-balance (S23-S24) conditions uniquely determine the solution ($c_C(r)$ and $c_O(r)$ distributions) for a given choice of radii, r_i, r_v, and r_e, enzyme concentrations, c_R and c_P, and specified exterior conditions.

The concentration of NAD-ME in the core can be calculated by noting that Φ_{C4} must match the total malate-decarboxylation rate, under conditions of saturation by malate,

$$c_N = \frac{\Phi_{C4}}{\frac{4\pi}{3} r_i^3 k_{catN}} \tag{S25}$$

S.2.4. Case of abundant PEPC

In the abundant PEPC region, minimal cost is achieved in the numerically unreachable $c_P \to \infty$ limit. To get an exact solution in this case, a modified problem is solved. Since now the entirety of PEP carboxylation occurs in vanishingly thin layers at $r = r_v$ and $r = r_e$, Equation S11 is simplified: $c_C\,(r_v < r < r_e) = 0$. The malate current is instead determined by the diffusive flow of CO_2 through the r_v and r_e boundaries,

$$\Phi_{C4} = 4\pi r_e^2 j_C - 4\pi r_v^2 D_C \left.\frac{\mathrm{d}c_C}{\mathrm{d}r}\right|_{r\to r_{v-}}, \tag{S26}$$

where j_C is given by[11] $j_C = D_C \left.\frac{\mathrm{d}c_C}{\mathrm{d}r}\right|_{r\to r_{e+}} = D_C c_{Ceq}/r_e$, if water is outside, or by $j_C = \sigma_B c_{Ceq}$, if air is outside. All other equations remain the same.

S.2.5. Expressions for CO_2 leakage and net assimilation rate

CO_2 leakage represents the part of the carbon delivered via the malate shuttle that subsequently escapes the core as CO_2. By its definition, it is a meaningful quantity only when the C_4 pump is active and the concentration of CO_2 within the core is larger than in the periphery. The escaping CO_2 current is

$$\Phi_{esc} = \Phi_{mitoC} - \Phi_C \tag{S27}$$

[11]The expression is obtained from $r\frac{\mathrm{d}}{\mathrm{d}r}c\,(r) + c\,(r) = c\,(r \to \infty)$ (see footnote 10), by setting $r = r_e$ and $c(r_e) = 0$.

The part of it that is due to malate decarboxylation (as opposed to photorespiration) is $\Phi_{esc}\,(\Phi_{C4}/\Phi_{mitoC})$. The relative leakage is then:

$$\phi_{leak} = \frac{\Phi_{esc}}{\Phi_{mitoC}} = 1 - \frac{\Phi_C}{\Phi_{C4} + \Phi_O/2} \tag{S28}$$

Expressions for the net assimilation rates per Rubisco and per unit volume are:

$$\text{assimilation per Rubisco} = \frac{\Phi_C - \Phi_O/2}{\frac{4\pi}{3} r_i^3 c_R} \tag{S29}$$

$$\text{assimilation per volume} = \frac{\Phi_C - \Phi_O/2}{\frac{4\pi}{3} r_e^3} \tag{S30}$$

S.3. Numerical implementation

The photon cost landscapes shown in the figures were evaluated on a grid of 101×101 points ($(r_i, r_v - r_i)$ pairs). For each point, differential equations (S.2) were solved using a standard shooting method, i.e. by varying the concentration of CO_2 and oxygen at the origin, $c_C(r = 0)$ and $c_O(r = 0)$, and their release rate in the core, ψ_{mitoC} and ψ_{chlorO}, so as to find a solution that (I) satisfies the boundary conditions at r_e (Eq. S16 and S17 or S14 and S15), and (II) satisfies the current balance conditions, Eq. S23 and S24. The equations were numerically integrated in the core region; beyond r_i the solution could be expressed analytically. The equations were solved for $c_P = 0$ and $c_P \to \infty$, to get the C_3 and abundant-PEPC solutions where possible. An adaptive sweep was also done across a range of finite c_P values for each point to find a finite-PEPC photon cost minimum (if present). This procedure was implemented in Python, using SciPy libraries for ODE integration and root finding (`scipy.integrate.odeint` and `scipy.optimize.fsolve`).

References

Cousins, A. B., Ghannoum, O., von Caemmerer, S., Badger, M. R., 2010. Simultaneous determination of Rubisco carboxylase and oxygenase kinetic parameters in *Triticum aestivum* and *Zea mays* using membrane inlet mass spectrometry. Plant, Cell & Environment 33 (3), 444–452.

Farquhar, G. D., von Caemmerer, S., Berry, J. A., 1980. A biochemical model of photosynthetic CO2 assimilation in leaves of C3 species. Planta 149 (1), 78–90.

Heinhorst, S., Williams, E. B., Cai, F., Murin, C. D., Shively, J. M., Cannon, G. C., 2006. Characterization of the Carboxysomal Carbonic Anhydrase CsoSCA from Halothiobacillus neapolitanus. Journal of Bacteriology 188 (23), 8087–8094.

Jenkins, C. L. D., Furbank, R. T., Hatch, M. D., 1989. Mechanism of C4 Photosynthesis A Model Describing the Inorganic Carbon Pool in Bundle Sheath Cells. Plant Physiology 91 (4), 1372–1381.

Johnson, K. S., 1982. Carbon dioxide hydration and dehydration kinetics in seawater1. Limnology and Oceanography 27 (5), 849–855.

Kai, Y., Matsumura, H., Inoue, T., Terada, K., Nagara, Y., Yoshinaga, T., Kihara, A., Tsumura, K., Izui, K., 1999. Three-dimensional structure of phosphoenolpyruvate carboxylase: A proposed mechanism for allosteric inhibition. PNAS 96 (3), 823–828.

Kramer, D. M., Evans, J. R., 2011. The Importance of Energy Balance in Improving Photosynthetic Productivity. Plant Physiology 155 (1), 70–78.

Mazarei, A. F., Sandall, O. C., 1980. Diffusion coefficients for helium, hydrogen, and carbon dioxide in water at 25 o C. AIChE Journal 26 (1), 154–157.

Reinfelder, J. R., Kraepiel, A. M. L., Morel, F. M. M., 2000. Unicellular C4 photosynthesis in a marine diatom. Nature 407 (6807), 996–999.

Rosnow, J. J., Evans, M. A., Kapralov, M. V., Cousins, A. B., Edwards, G. E., Roalson, E. H., 2015. Kranz and single-cell forms of C4 plants in the subfamily Suaedoideae show kinetic C4 convergence for PEPC and Rubisco with divergent amino acid substitutions. Journal of Experimental Botany, 66, 7347-7358.

Sage, R. F., 2004. The evolution of C $_4$ photosynthesis. New Phytologist 161 (2), 341–370.

Tholen, D., Zhu, X.-G., 2011. The Mechanistic Basis of Internal Conductance: A Theoretical Analysis of Mesophyll Cell Photosynthesis and CO2 Diffusion. Plant Physiology 156 (1), 90–105.

Tronconi, M. A., Fahnenstich, H., Weehler, M. C. G., Andreo, C. S., Flügge, U.-I., Drincovich, M. F., Maurino, V. G., 2008. Arabidopsis NAD-Malic Enzyme Functions As a Homodimer and Heterodimer and Has a Major Impact on Nocturnal Metabolism. Plant Physiology 146 (4), 1540–1552.

von Caemmerer, S., Edwards, G. E., Koteyeva, N., Cousins, A. B., 2014. Single cell C4 photosynthesis in aquatic and terrestrial plants: A gas exchange perspective. Aquatic Botany 118, 71–80.

von Caemmerer, S., Furbank, R. T., 2003. The C4 pathway: an efficient CO2 pump. Photosynthesis Research 77 (2-3), 191–207.
Walker, N. A., Smith, F. A., Cathers, I. R., 1980. Bicarbonate assimilation by fresh-water charophytes and higher plants: I. Membrane transport of bicarbonate ions is not proven. Journal of Membrane Biology 57 (1), 51–58.
Zhu, X.-G., Long, S. P., Ort, D. R., 2010. Improving Photosynthetic Efficiency for Greater Yield. Annual Review of Plant Biology 61 (1), 235–261.
