# Supplementary material for: Size matters for single-cell C4 photosynthesis in Bienertia
Source: J Exp Bot. 2016 Oct 12;68(2):255–67. doi: 10.1093/jxb/erw374 (PMC5853773; doi:10.1093/jxb/erw374)
Supplement: Supplementary_Table_S1_Figures_S1_S14 [file erw374_suppl_supplementary_table_s1_figures_s1_s14.pdf]

Size matters for single-cell C_4 photosynthesis in *Bienertia*: Supplementary material (figures and tables)

Ivan Jurić[a,b], Vinicio González-Pérez[a], Julian M. Hibberd[c], Gerald Edwards[d], Nigel J. Burroughs[a]

[a] *Warwick Systems Biology Centre, University of Warwick, Coventry, CV4 7AL, UK*
[b] *Institute of Physics, Bijenička c. 46, P.O. Box 304, HR-10001 Zagreb, Croatia*
[c] *Department of Plant Sciences, University of Cambridge, Cambridge, CB2 3EA, UK*
[d] *School of Biological Sciences, Washington State University, Pullman, WA 99164-4236, USA*

Table S1: Parameter values for modelling C_4 photosynthesis at different temperatures. Only the parameters that change with temperature are listed; others are assumed temperature invariant. The temperature response of Rubisco kinetic parameters was estimated by taking the values for *Z. mays* at 20°C (Cousins et al., 2010), and calculating the values at different temperatures by assuming the parameteres follow an activated behaviour with the same activation energies as in *S. viridis* (Boyd et al., 2015). PEPC and NAD-ME kinetic parameters were not altered because the changes in their activities, unless drastic, will not affect the optimal photon cost, since the necessary C_4 acid flow can be sustained simply by changing the enzyme concentrations. Solubility of atmospheric gases at different temperatures has been thoroughly investigated, and we take our values for c_{Ceq} and c_{Oeq} from (Carroll et al., 1991; Murray and Riley, 1969). Both O_2 and CO_2 solubilities drop with a rise in temperature, but this drop is more pronounced in the case of CO_2, presenting a further challenge for hot climate plants. For the diffusion constant and barrier permeability of dissolved gases, we assume a linear dependence on absolute temperature (Mostinsky, 2006), which will exert only a marginal influence on the resulting costs, since the other parameters display a stronger (Arrhenius-type) dependence.

Symbol	Units	Value at 10°C	Value at 20°C	Value at 30°C	Value at 40°C
k_{catC}	s^{-1}	1.51	4.7	13.6	36.6
k_{catO}	s^{-1}	0.22	0.49	1.04	2.10
K_C	μM	6.36	16.2	38.8	87.8
K_O	μM	157	183	211	241
c_{Ceq}	μM	21.0	15.4	11.6	8.95
c_{Oeq}	μM	353	284	236	202
D_C, D_O	$\mu m^2/s$	1739	1800	1861	1923
σ_B	μm/s	96.6	100	103	107

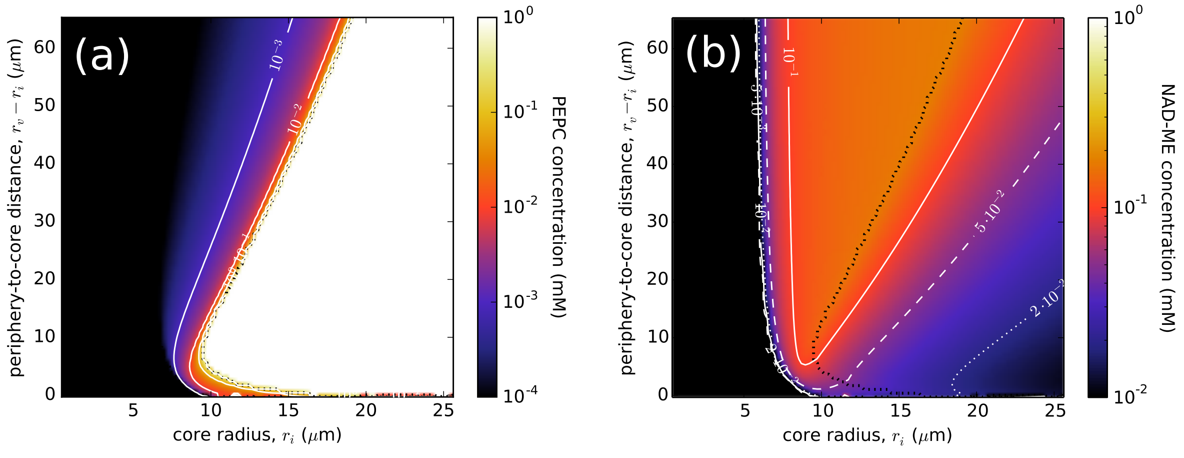

Figure S1: The optimal concentrations (in mM) of PEPC in the periphery (a) and NAD-ME in the core region (b), as functions of r_i and $r_v - r_i$. The abundant PEPC region, which appears white in (a), has PEPC concentration above 1 mM; the black region on the left is the C_3 region, with zero PEPC concentration. Dotted black lines show the boundaries between different PEPC regime regions. Panel (a) is repeated from Fig. 2(a). Parameter values are as in Fig. 2. For details of NAD-ME concentration calculation see Sec. **??**.

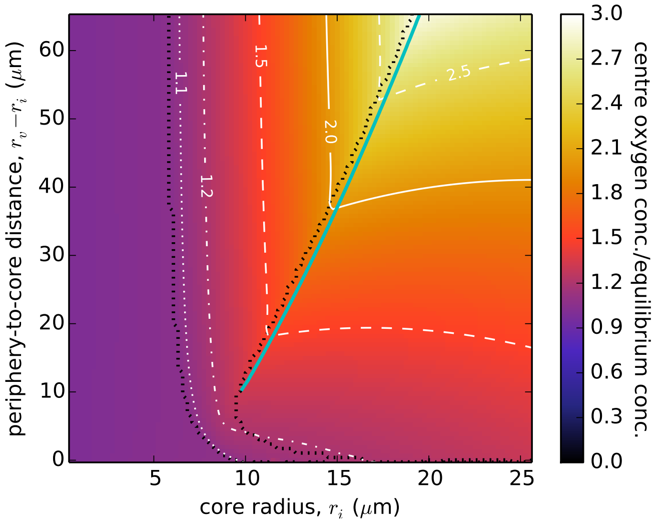

Figure S2: Oxygen concentration in the cell centre relative to its equilibrium dissolved concentration (i.e. the concentration of dissolved oxygen at equilibrium with its partial pressure in surrounding air), $c_O(r = 0)/c_{Oeq}$. Parameters as in Fig. 2 and 3.

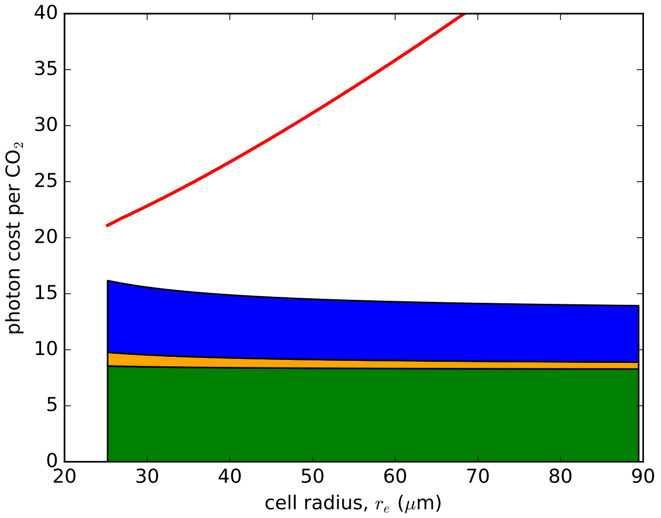

Figure S3: Breakdown of the C_4-optimised photon cost along the optimal-geometry line of Fig. 3, as a function of the cell radius, r_e. Calvin-Benson cycle cost is in green, photorespiration cost in orange, and C_4 pump cost in blue. The red line shows the C_3 pathway photon cost for the same cell geometry. Parameters as in Fig. 2 and 3.

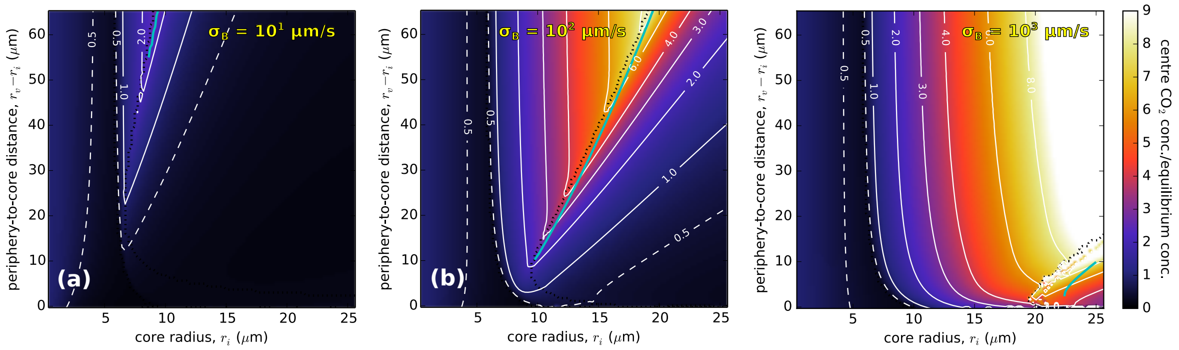

Figure S4: Concentrations of CO_2 in the cell centre for three values of the cell boundary permeability. (a): σ_B = 10 µm/s; (b): $\sigma_B = 10^2$ µm/s (as Fig. 6(a)); (c): $\sigma_B = 10^3$ µm/s. Corresponding photon cost landscapes are shown in Fig. 8. Lines as in Fig. 6(a). Parameters as in Fig. 8.

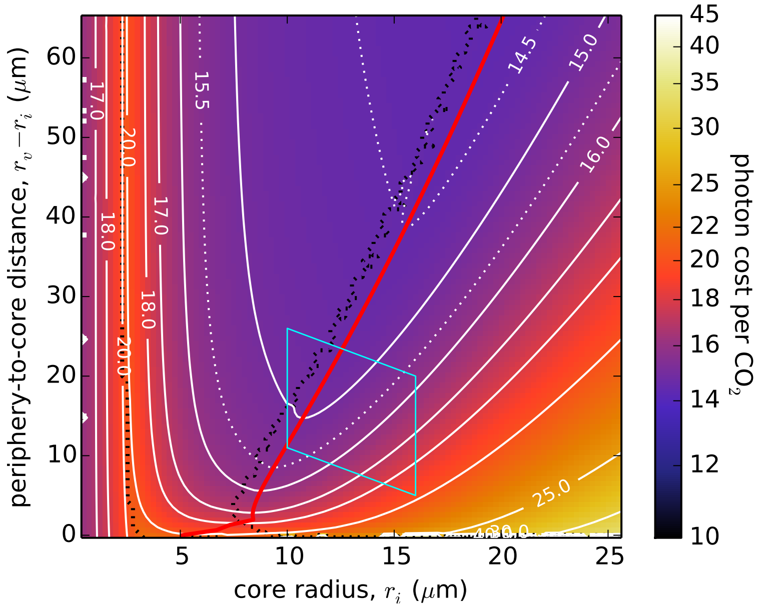

Figure S5: Photon cost landscape at 40°C with a cell barrier permeability $\sigma_B = 10^3$ µm/s. Lines as in Fig. 3(a). Parameters in Table S1. The scatter of the finite-abundant PEPC border line is due to numerical limitations of the solver at these extreme parameter values.

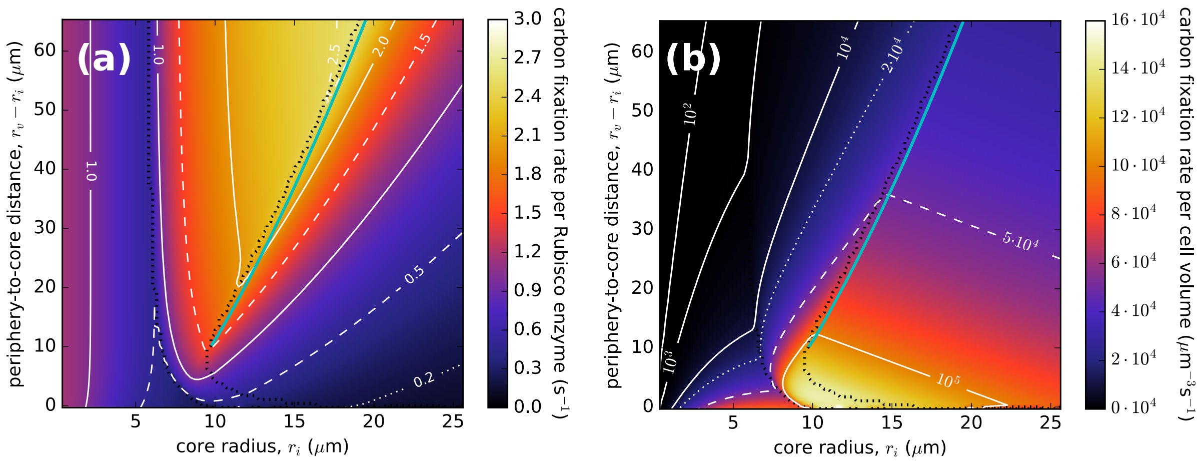

Figure S6: Net carbon assimilation rate per (a) Rubisco enzyme (in s^{-1}), and per (b) cell volume (in $\mu m^{-3} s^{-1}$), as a function of r_i and $r_v - r_i$. The region of high per-Rubisco assimilation rate matches the location of the photon cost valley in Fig. 3. Parameters as Fig. 2.

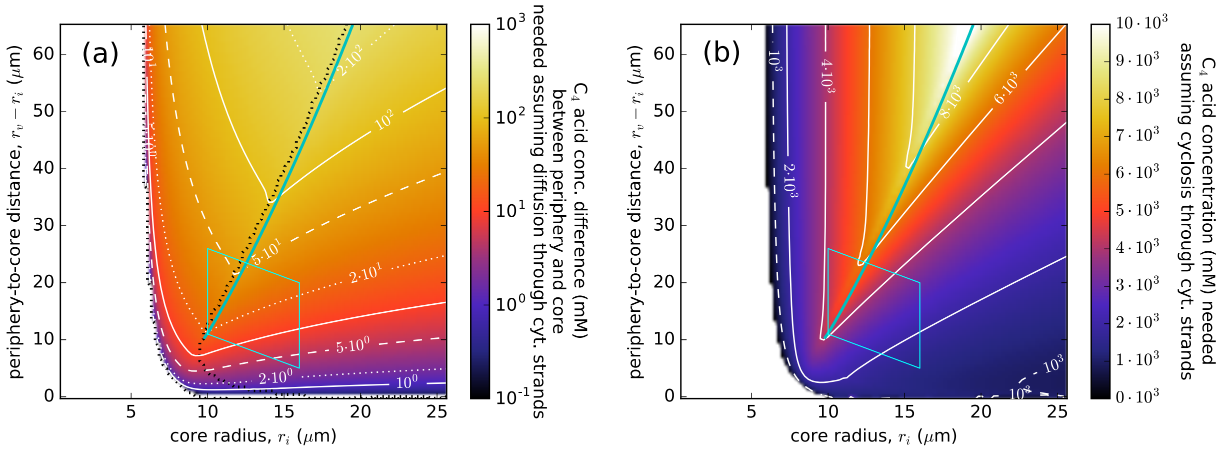

Figure S7: C_4 acid concentration levels needed to drive the C_4 pump. Panel (a): C_4 acid concentration difference (in mM) between the periphery and the core needed to drive the optimally performing C_4 pump for a given cell geometry. For optimal functioning of the C_4 pump, the concentrations of C_3 acids in the periphery and of C_4 acids in the core have to be high enough to saturate the respective enzymes (around 30 mM). However, this requires the concentration of the acids at their source to be even higher, since a concentration difference is needed to drive the diffusion current between the two compartments. This difference is likely to be much greater, in absolute terms, than it is for the case of gases considered in the model (Fig. 5), because the transport of C_3 and C_4 acids is likely constrained to narrow cytoplasmic strands that connect the core and the periphery. In the following, we assume that only 1% of CCC surface area is adjacent to open cytoplasmic strands. This is a *very* conservative estimate, and images obtained by scanning electron microscopy would suggest the percentage is likely higher (Voznesenskaya et al., 2005). Panel (a) shows the difference (in mM) between the C_4 acid concentration in the periphery and in the core needed to drive the optimally performing C_4 pump for a given cell geometry. The difference is obtained by equating the total C_4 current, Φ_{C4}, with the diffusive flow of C_4 acid through thin strands, $DA\left(c_{periphery} - c_{core}\right) / (r_v - r_i)$, where A is the total cross-section area of cytoplasmic strands $\left(A = 1\% \cdot 4\pi r_i^2\right)$, and the diffusion constant D is set to 1000 $\mu m^2/s$, a value appropriate for small organic molecules. The difference in Fig. (a) is in the 100 mM range for large cores and core-to-periphery separations, but the concentration difference stays between 5 mM and 50 mM within the region of observed Bienertia cell sizes (light-blue rectangle). This *relatively* modest concentration gradient is both sufficient and necessary to drive the metabolite currents; cyclosis alone (with a mean speed of 0.8 μm/s (Nebenführ et al., 1999)) cannot produce the required flow for metabolite concentrations (within the strands) below 4 M. Panel (b) shows the necessary C_3 and C_4 acid concentration in the strands for transport by cyclosis; it is obtained by equating Φ_{C4} with the cyclosis current $v_{cyclosis} A c_{strand}$. Parameters as in Fig. 2.

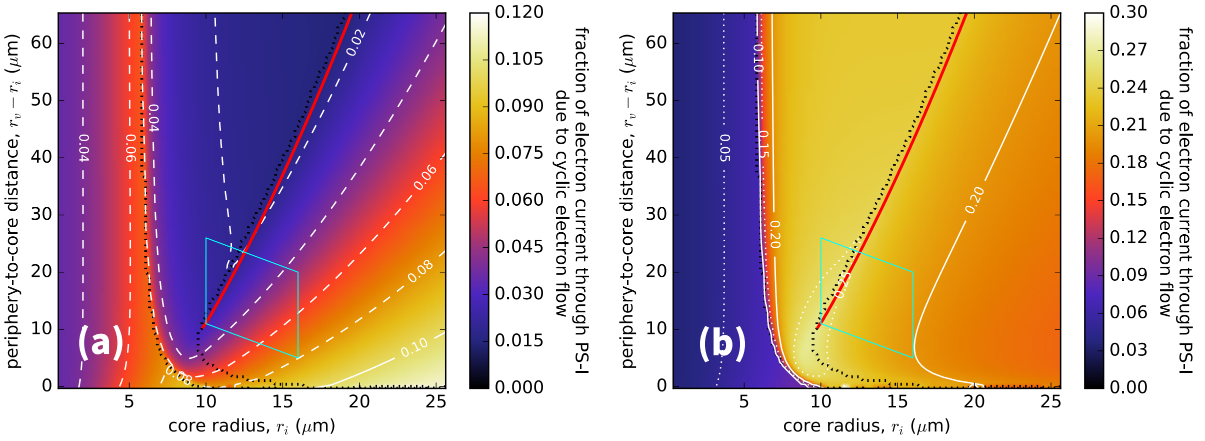

Figure S8: Fraction of the electron current passing through photosystem-I that is due to the cyclic electron flow (the remainder constituting linear electron transfer). (a): Cyclic flow fraction in the core chloroplasts assuming the core provides NADPH and ATP *exclusively* to Calvin-Benson and photorespiratory cycles. This is consistent with observations that peripheral chloroplast are deficient in photosystem-II (Voznesenskaya et al., 2002). In this case, peripheral chloroplasts would produce ATP needed for the C_4 pump operation, and the cyclic flow fraction in the periphery would be 100%. (b): Overall cyclic flow fraction in a Bienertia cell (core and periphery chloroplasts combined). In both figures we ignore the ATP/NADPH requirements of other cell processes. The cell needs ATP and reducing power for other vital functions, so the actual cyclic flow fraction depends on the overall ATP/NADPH use ratio. Parameters as in Fig. 2.

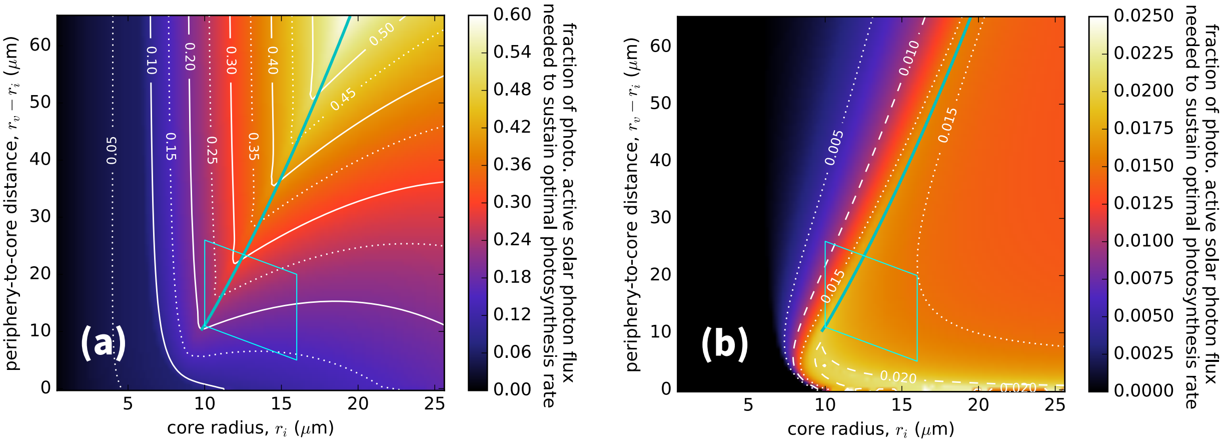

Figure S9: Photosynthetically active photon flux (PAPF) needed to sustain photosynthesis. (a): PAPF incident upon a Bienertia cell's core compartment needed to sustain energy requirements of the Calvin-Benson cycle and photorespiration. (b): PAPF incident upon a Bienertia cell needed to sustain energy requirements of the C_4 pump operation. Fluxes are expressed as fractions of the total solar PAPF incident upon the Earth's surface. For this quantity a conservative estimate of peak sunlight, $1.2 \cdot 10^9$ $\mu m^{-2}s^{-1}$ = 2 mmol/m^2s, is used (Björkman and Demmig-Adams, 1995). Fluxes are evaluated by weighting the overall Rubisco carboxylation, oxygenation, and the C_4 current with the respective photon costs and dividing by the cross-sectional area of a Bienertia cell or its core, i.e. $(\varphi_C \Phi_C + \varphi_O \Phi_O)/\pi r_i^2$ in (a), and $\varphi_{C4}\Phi_{C4}/\pi r_e^2$ in (b). This is a very crude way to estimate the light requirements. We assume, as in Fig. S8, that core chloroplasts provide ATP and NADPH for the Calvin-Benson cycle and photorespiration, while the peripheral chloroplasts only provide ATP for the C_4 pump operation. Parameters as Fig. 2. Note that, because of the light absorption by the leaf tissue as well as the possible light saturation of chloroplast photosystems (Zhu et al., 2010), even a 10% fraction represents a significant fraction. If the peripheral chloroplasts contribute to NADPH production, the 'burden' on the core chloroplast photosystems may be substantially lighter. Peripheral chloroplasts have reduced grana development, suggestive of an increased capacity for cyclic electron flow (Voznesenskaya et al., 2002). However, they also possess some capacity to reduce 3-phosphoglyceric acid (PGA) by linear electron flow (Offermann et al., 2011), which suggests they may support the reductive phase of the Calvin-Benson cycle by exchanging metabolites between the core and the periphery.

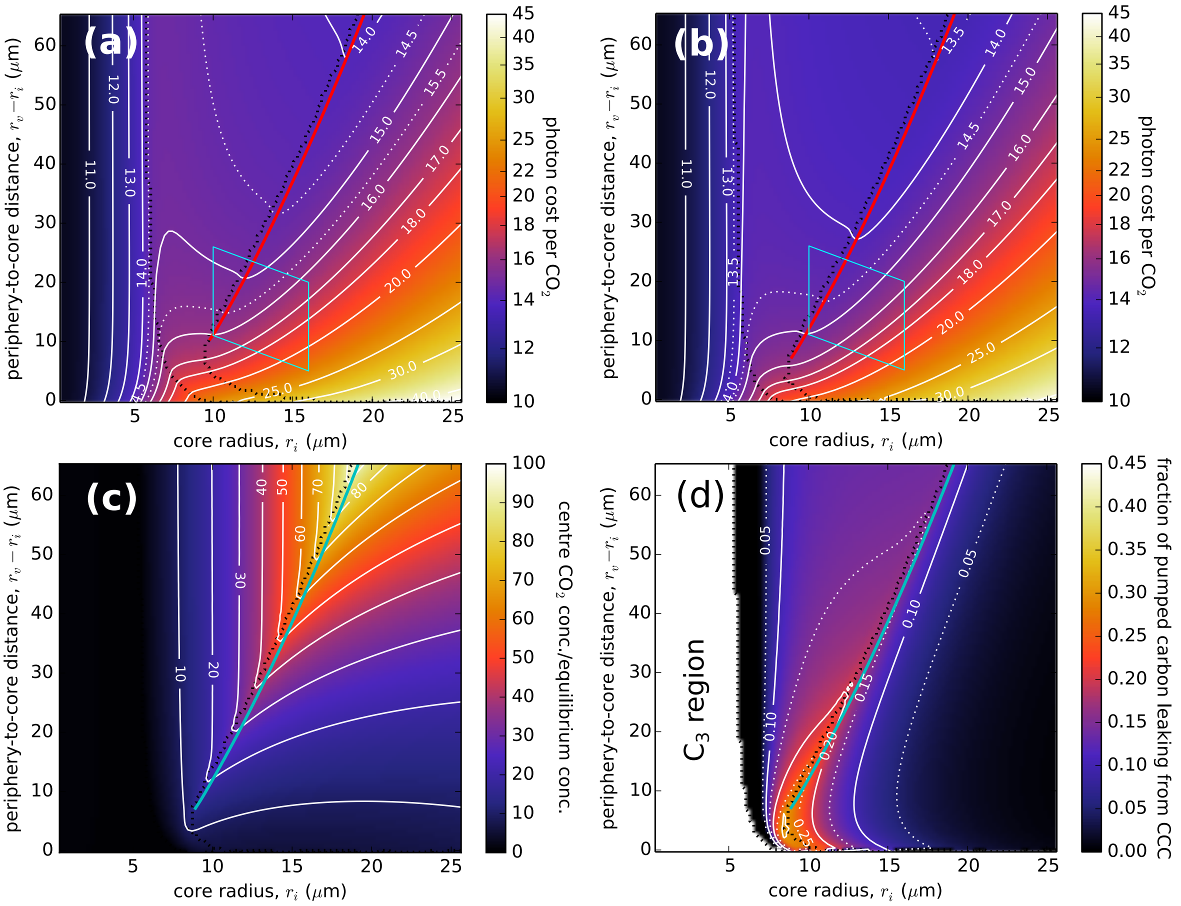

Figure S10: Repositioning mitochondria at the centre of the core region. (a) and (b): Comparison of photon-cost landscapes in the cases where (a) mitochondria are spread throughout the core region (as Fig. 3) and where (b) they are concentrated within a sphere, with radius equal to one third of r_i, at the cell centre. Lines and parameter values as in Fig. 3. (c): CO_2 concentration in the centre relative to the external dissolved concentration, $c_C(r=0)/c_{Ceq}$, in the case of concentrated mitochondria. (d): CO_2 leakage from the core in the case of concentrated mitochondria. Lines as in Fig. 6. Other parameters as Fig. 2.

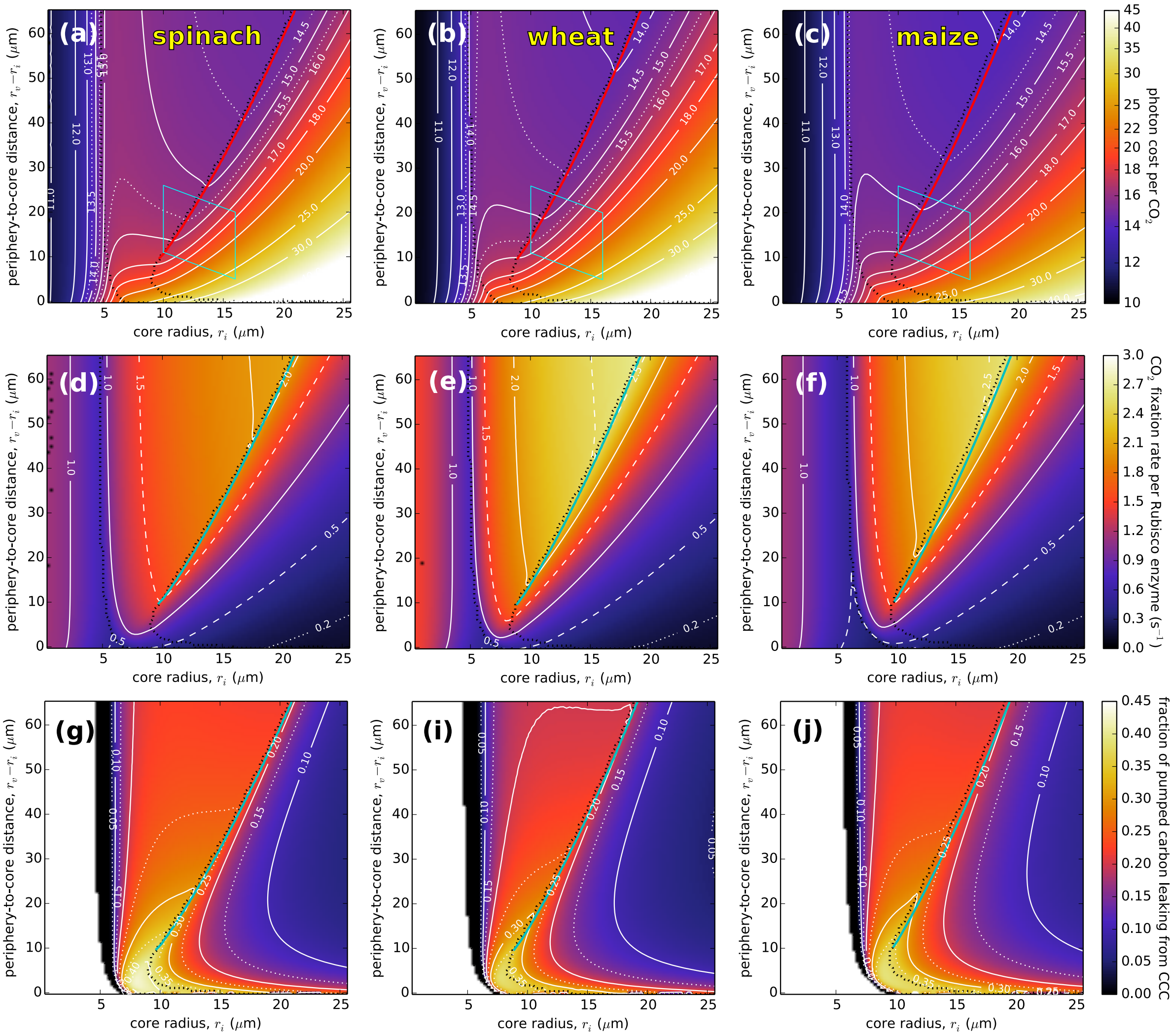

Figure S11: Comparison of C_4 pump efficiency for differing Rubisco enzyme characteristics. Left column: spinach (*Spinacea oleracea*) Rubisco; middle column: wheat (*Triticum aestivum*) Rubisco; right column: maize (*Zea mays*) Rubisco. Spinach and wheat are C_3 plants, while maize is a C_4 plant. (a)-(c): photon cost landscapes. (d)-(f): Corresponding per-Rubisco assimilation rates. (g)-(j): CO_2 leakage. Kinetic properties were taken from (Zhu et al., 1998) and (Cousins et al., 2010). Specifically, for spinach: k_{catC} = 2.7 s^{-1}, k_{catO} = 1.5 s^{-1}, K_C = 11 µM, K_O = 520 µM; for wheat: k_{catC} = 3.8 s^{-1}, k_{catO} = 0.83 s^{-1}, K_C = 9.7 µM, K_O = 244 µM; for maize: see Table 1. Other parameters as Fig. 2.

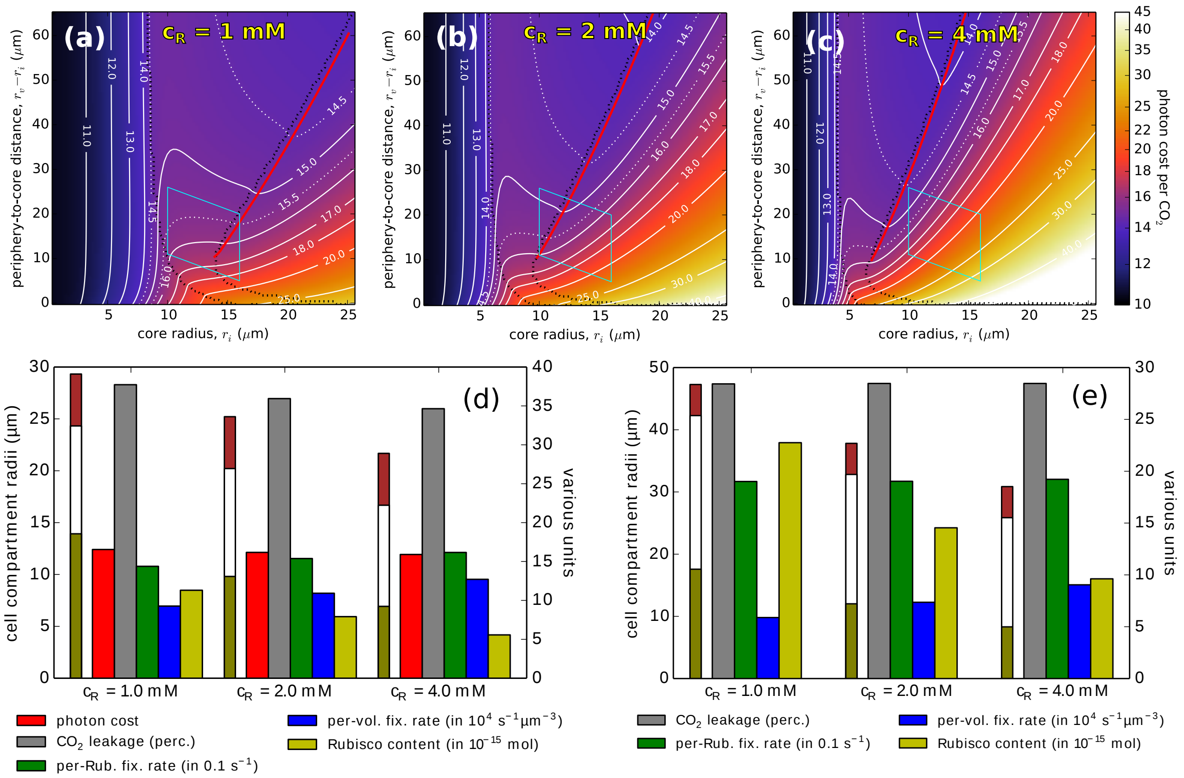

Figure S12: Varying the Rubisco concentration in the core. (a)-(c): The C_4-optimised photon cost landscapes for various Rubisco active site concentrations: c_R = 1, 2 (repeated from Fig. 3(a)), and 4 mM. Lines as Fig. 3(a). (d): Comparison of CO_2 leakage, photon cost, assimilation rates, total core Rubisco (active site) content, and the cell dimensions at the starting points of the optimal-geometry lines in figures (a) to (c). The three compartment radii are marked on the left-most tricolour meters, and can be read on the left y-axis. The values of other quantities are read on the right y-axis. The total amount of Rubisco in the core is lower at higher Rubisco concentrations, as the increase in concentration leads to a shift in the onset of the C_4 region to smaller core radii and smaller periphery-to-core distances (compare panels (a)-(c)). Even so, the photon costs and per-Rubisco assimilation rates remain close in value indicating that the C_4 pump would run with near identical efficacy. However, there is a notable difference in the net carbon assimilation rate per unit volume, which is higher in cells with a higher density of Rubisco in the CCC. (e): A comparison taken at the points on the optimal-geometry lines where the C_4-optimised photon cost equals 15 photons per carbon. The CO_2 leakage is also equal at those points; this is because, under conditions when photorespiration is effectively extinguished, CO_2 leakage and the photon cost will both depend only on the activity ratio of the PEP and RuBP carboxylases. Parameters as Fig. 2

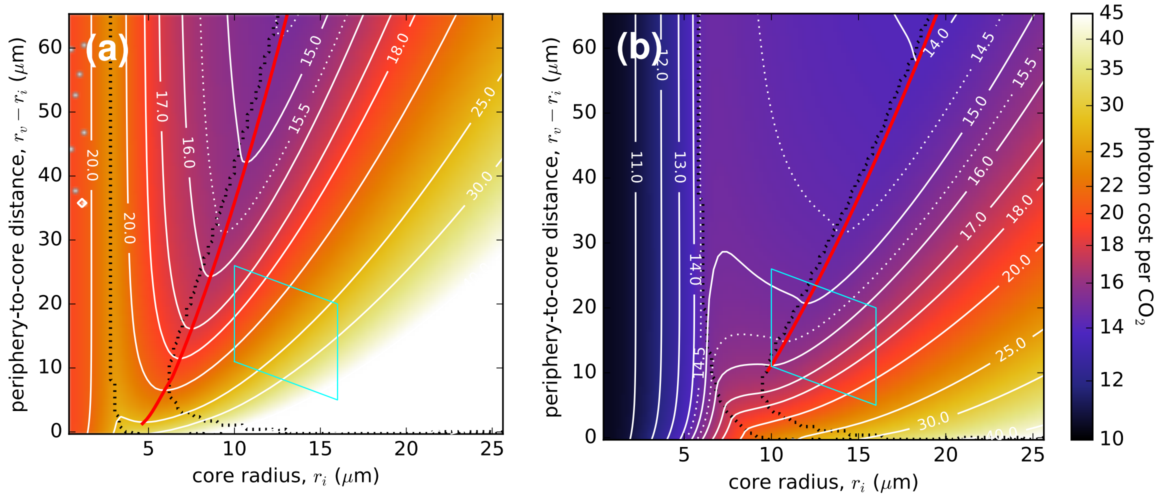

Figure S13: Varying the ambient CO_2 concentration. (a): The photon cost landscape when the concentration of CO_2 outside the cell is set at 120 ppm (30% of its current atm. value). (b): The photon cost landscape at 400 ppm external CO_2 concentration (same as Fig. 3). Other parameters as Fig. 2. The photon cost is higher at low CO_2 concentration, and the valley in the C_4 region is much deeper. The onset of the C_4 region appears at smaller core radii, and the photon cost in the C_3 region is higher (in particular, it is higher than in the valley region).

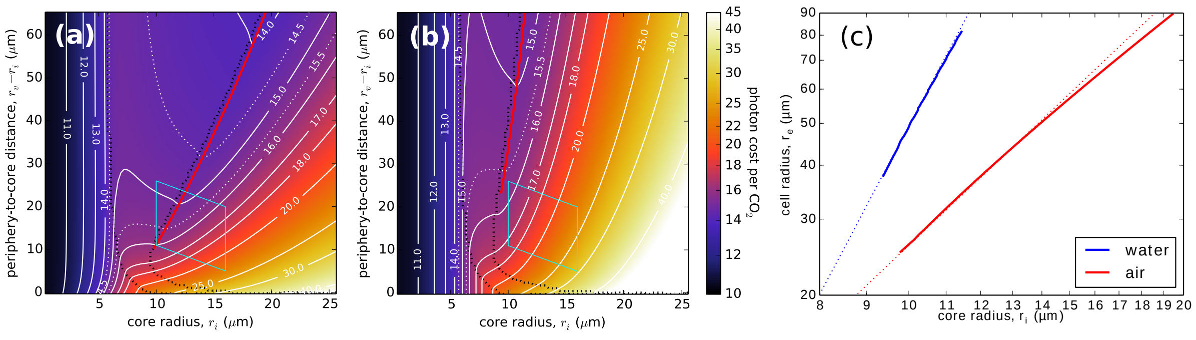

Figure S14: Comparison of water and air environments. (a) and (b): C_4-optimised photon cost landscapes in air (repeated from Fig. 3(a)) and water environments. Lines and parameter values as in Fig. 3(a). (c): Core (r_i) and cell (r_e) radii along the optimal-geometry lines in the two environments, plotted on a log-log scale. Dotted lines show slopes with inclinations of 2 (red) and 4 (blue). The scaling between the core size and the cell size along the optimal-geometry line is determined by the balance between C_4 pump costs and photorespiration costs (see discussion in Sec. 3.2). The figure shows the two radii roughly scaling as $r_i \sim r_e^{1/2}$ in air and $r_i \sim r_e^{1/4}$ in water, which, along with fact that the CO_2 intake is absorption limited in air and diffusion limited in water (scaling as r_e^2 and r_e^1 respectively), suggests that the *effective* carboxylation capacity of the core grows approximately as r_i^4.

References

Björkman, O., Demmig-Adams, B., 1995. Regulation of Photosynthetic Light Energy Capture, Conversion, and Dissipation in Leaves of Higher Plants. In: Schulze, P. D. E.-D., Caldwell, P. D. M. M. (Eds.), Ecophysiology of Photosynthesis. No. 100 in Springer Study Edition. Springer Berlin Heidelberg, pp. 17–47, dOI: 10.1007/978-3-642-79354-7_2.

Boyd, R. A., Gandin, A., Cousins, A. B., 2015. Temperature Responses of C4 Photosynthesis: Biochemical Analysis of Rubisco, Phosphoenolpyruvate Carboxylase, and Carbonic Anhydrase in Setaria viridis. Plant Physiology 169 (3), 1850–1861.

Carroll, J. J., Slupsky, J. D., Mather, A. E., 1991. The Solubility of Carbon Dioxide in Water at Low Pressure. Journal of Physical and Chemical Reference Data 20 (6), 1201.

Cousins, A. B., Ghannoum, O., von Caemmerer, S., Badger, M. R., 2010. Simultaneous determination of Rubisco carboxylase and oxygenase kinetic parameters in *Triticum aestivum* and *Zea mays* using membrane inlet mass spectrometry. Plant, Cell & Environment 33 (3), 444–452.

Mostinsky, I., 2006. Diffusion coefficient. In: A-to-Z Guide to Thermodynamics, Heat and Mass Transfer, and Fluids Engineering: AtoZ. Vol. D. Begellhouse.

Murray, C. N., Riley, J. P., 1969. The solubility of gases in distilled water and sea water—II. Oxygen. Deep Sea Research and Oceanographic Abstracts 16 (3), 311–320.

Nebenführ, A., Gallagher, L. A., Dunahay, T. G., Frohlick, J. A., Mazurkiewicz, A. M., Meehl, J. B., Staehelin, L. A., 1999. Stop-and-Go Movements of Plant Golgi Stacks Are Mediated by the Acto-Myosin System. Plant Physiology 121 (4), 1127–1141.

Offermann, S., Okita, T. W., Edwards, G. E., 2011. Resolving the Compartmentation and Function of C4 Photosynthesis in the Single-Cell C4 Species Bienertia sinuspersici. Plant Physiology 155 (4), 1612–1628.

Voznesenskaya, E. V., Franceschi, V. R., Kiirats, O., Artyusheva, E. G., Freitag, H., Edwards, G. E., 2002. Proof of C4 photosynthesis without Kranz anatomy in Bienertia cycloptera (Chenopodiaceae). The Plant Journal 31 (5), 649–662.

Voznesenskaya, E. V., Koteyeva, N. K., Chuong, S. D. X., Akhani, H., Edwards, G. E., Franceschi, V. R., 2005. Differentiation of cellular and biochemical features of the single-cell C4 syndrome during leaf development in Bienertia cycloptera (Chenopodiaceae). American Journal of Botany 92 (11), 1784–1795.

Zhu, G., Jensen, R. G., Bohnert, H. J., Wildner, G. F., Schlitter, J., 1998. Dependence of catalysis and CO2/O2 specificity of Rubisco on the carboxy-terminus of the large subunit at different temperatures. Photosynthesis Research 57 (1), 71–79.

Zhu, X.-G., Long, S. P., Ort, D. R., 2010. Improving Photosynthetic Efficiency for Greater Yield. Annual Review of Plant Biology 61 (1), 235–261.
